# Defining the genetics of the widely used G3 strain of the mosquito, *Anopheles gambiae*

**DOI:** 10.1038/s41598-025-96391-y

**Published:** 2025-04-16

**Authors:** Melina Campos, Marc Crepeau, Gregory C. Lanzaro

**Affiliations:** Vector Genetics Laboratory, Department of Pathology, Microbiology and Immunology, UC Davis, Davis, CA 95616 USA

**Keywords:** Malaria, Mosquito, Laboratory colony, Genetics, Evolution, Genetics

## Abstract

Mosquito species in the *Anopheles gambiae* complex have been referred to as “the deadliest animals in the world” due to their role as vectors of malaria throughout sub-Saharan Africa. Consequently, *An. gambiae* was among the first species to have its whole genome sequenced in 2002 and it continues to be the subject of intense study. *An. gambiae* is one member of a nine member species complex and, along with its sister species, *An. coluzzii*, is among the most important vectors of human malaria. Laboratory research on malaria vectors across a broad range of disciplines utilizes a strain known as G3, which was established in 1975 from mosquitoes collected from McCarthy Island, The Gambia. This strain is well known to be a mongrel strain, nonetheless it is often referred to as *An. gambiae*, which it is not. The issue with G3 goes far beyond the typical inbreeding associated with long-standing laboratory colonies. G3 is an *An. gambiae*/*An. coluzzii* interspecific hybrid. Although these two species are known to hybridize in nature, the pattern of interspecific introgression in G3 we describe in this paper is unlike any observed in natural populations. In this report we provide an in-depth analysis of the genetics of the G3 strain and compare it with natural populations of its two parental species. We discuss potential concerns that results obtained from research using the G3 strain may not apply to populations of these mosquito species as they occur in nature.

**Supplementary Information:**

The online version contains supplementary material available at 10.1038/s41598-025-96391-y.

## Introduction

Laboratory strains of insects have been instrumental in entomological research for decades, offering valuable insights into insect biology. One of the primary advantages of using these strains is that they are generally inbred, resulting in consistent and predictable traits that can be reliably studied and replicated. Additionally, laboratory strains are typically easy to rear and maintain, with well-established protocols that make them cost-effective and convenient for controlled experiments. However, there are also notable disadvantages. One major concern is that laboratory strains may not accurately represent the genetic and phenotypic diversity present in the natural insect populations from which they were established and are assumed to model^1,2^. Laboratory strains typically diverge from natural populations due to founder effects at the time they are established and repeated bottlenecks while being maintained and sub colonized over time. Genetic drift and selection for adaptation to the unnatural laboratory environment are the forces that drive this divergence. Ultimately, laboratory strains may exhibit reduced fitness if introduced back into the natural environment^3^, for example, as part of a biological or genetic-based insect control scheme. In *An. gambiae,* a transcriptome analysis comparing natural (field collected) and laboratory colony individuals revealed significant divergence between the two groups, with wild mosquitoes showing elevated expression of genes involved in insecticide resistance, immunity, and olfaction, while genes related to metabolism and protein synthesis were more highly expressed in colonized populations^4^.

The mosquito, *Anopheles gambiae*, is one of the most important insect species with respect to its impact on mankind. It is a major vector of human malaria, a disease which has played a significant role in both the cultural and biological evolution of mankind. Consequently, *An. gambiae* is one of the most intensively studied animal species. Most of this research has focused on aspects of its biology involved with malaria transmission. This research has relied heavily on a specific laboratory strain known as the G3 strain. Indeed, much of what we know about malaria biology has been derived from research using this strain. These studies include mosquito reproductive biology^5–7^, insecticide resistance^8–10^, malaria parasite/vector interactions^11–16^, salivary proteins as potential transmission blocking vaccines^17–19^ and the mosquito immune system in general^20,21^.

The G3 strain was first established from mosquitoes collected at McCarthy Island, The Gambia in 1975 and has since been maintained in laboratories worldwide. It is one of the strains distributed to researchers by the Malaria Research and Reference Reagent Center (MR4; BEI Resources). This nearly 50-year-old strain has been shown to be amenable to genetic manipulation. Recently, G3 has been widely used to develop new tools for controlling mosquito populations and reducing malaria transmission through genetic engineering^22–28^. For genetic engineering involving gene-drive systems, the genetic background of the target mosquito population may play an important role^24,25^, raising concerns about the G3 strain’s potential lack of similarity to natural populations. However, some studies have demonstrated that gene drive systems can be successfully introduced into different mosquito strains without a reduction in efficiency. Nevertheless, researchers should prioritize introducing these systems into target populations, such as recently established field populations, to ensure their effectiveness in real-world settings^25,29,30^.

*Anopheles gambiae* is the nominal species in a complex of nine closely related species^31^. Initially, the G3 strain was considered to be *An. gambiae* sensu stricto (hereafter *An. gambiae*), but currently, it has been confirmed to be a hybrid strain between the sister species *An. gambiae* and *An. coluzzii* (BEI Resources^24,32^). High rates of hybridization between these two species have been reported in The Gambia, where the G3 strain originated, and in Guinea-Bissau^33–36^, but in most localities, hybrids are rare making up less than 1% of individuals^37^. In this study, we investigate the genomics of the G3 strain in relation to field-collected populations of *An. gambiae* and *An. coluzzii* across Africa. Descriptions of mosquito colony genetics have been published for other mosquito species and other laboratory strains of *An. gambiae*^38–42^, but the only study of G3, to our knowledge, was conducted by Norris et al.^43^ who genotyped 9 microsatellite markers on chromosome 3 and found an 8-fold reduction in the mean number of alleles and a 3.5-fold reduction in mean heterozygosity. The goal of this study is to conduct a more thorough examination of the G3 strain using a comparative genomics approach to explore its relationship to natural populations of *An. coluzzii* and *An. gambiae* and to compare sub-colonies of G3 in use in two research laboratories. This analysis is significant because the G3 strain has been, and continues to be, the exemplar for important facets of malaria vector biology, even though it is likely an artificial population quite distinct from any that exist in nature.

## Results

### Population genomics

**Fig. 1:**
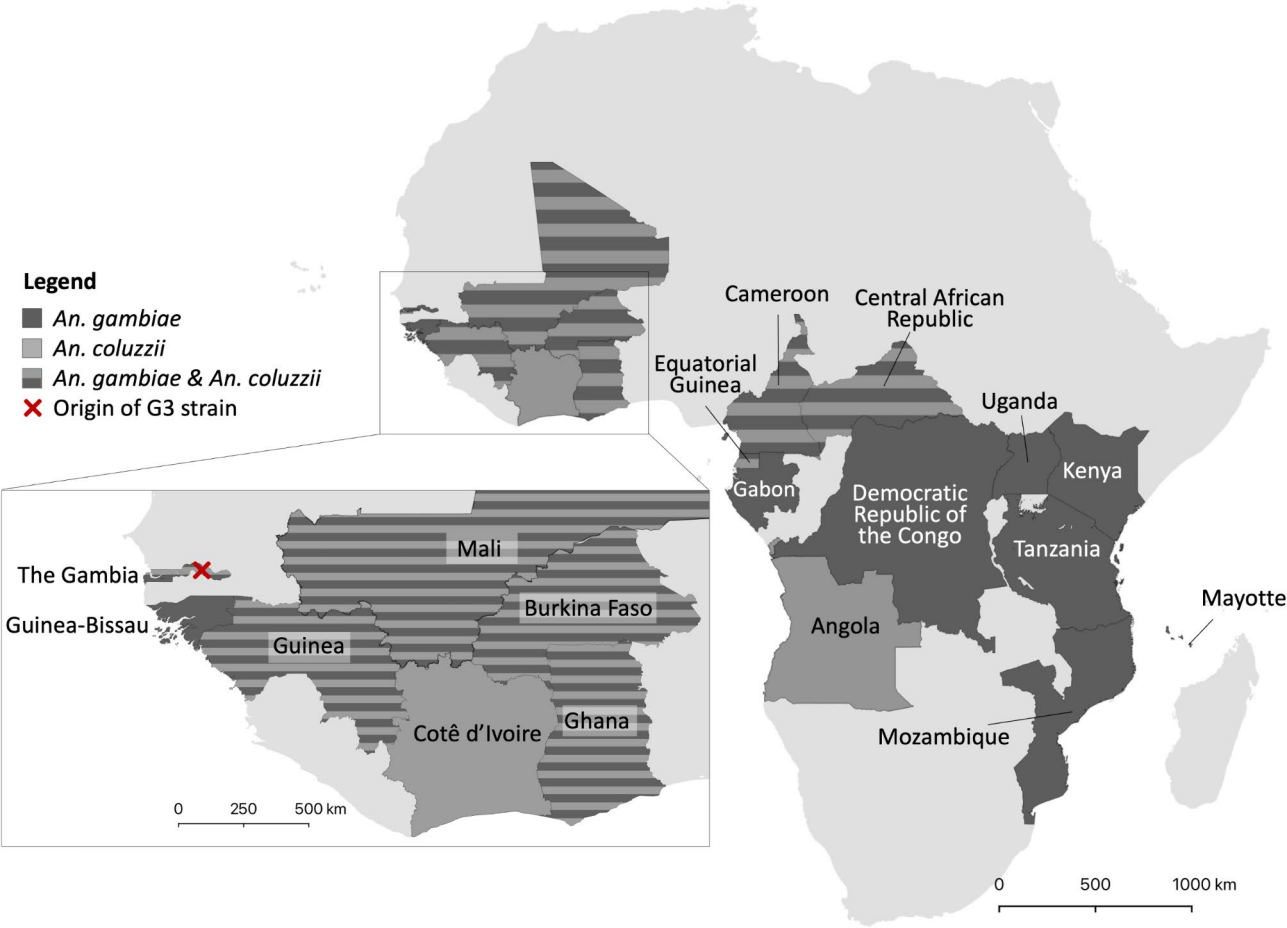
Population sampling. Whole genome sequencing data was obtained for samples of *Anopheles gambiae* (dark shade) and *An. coluzzii* (light shade) or both (striped) from 17 countries in continental Africa: Angola, Burkina Faso, Cameroon, Central African Republic, Cotê d’Ivoire, Democratic Republic of the Congo, Equatorial Guinea, Gabon, Ghana, Guinea, Guinea-Bissau, Kenya, Mali, Mozambique, Tanzania, The Gambia, Uganda; and Mayotte Island.

**Fig. 2:**
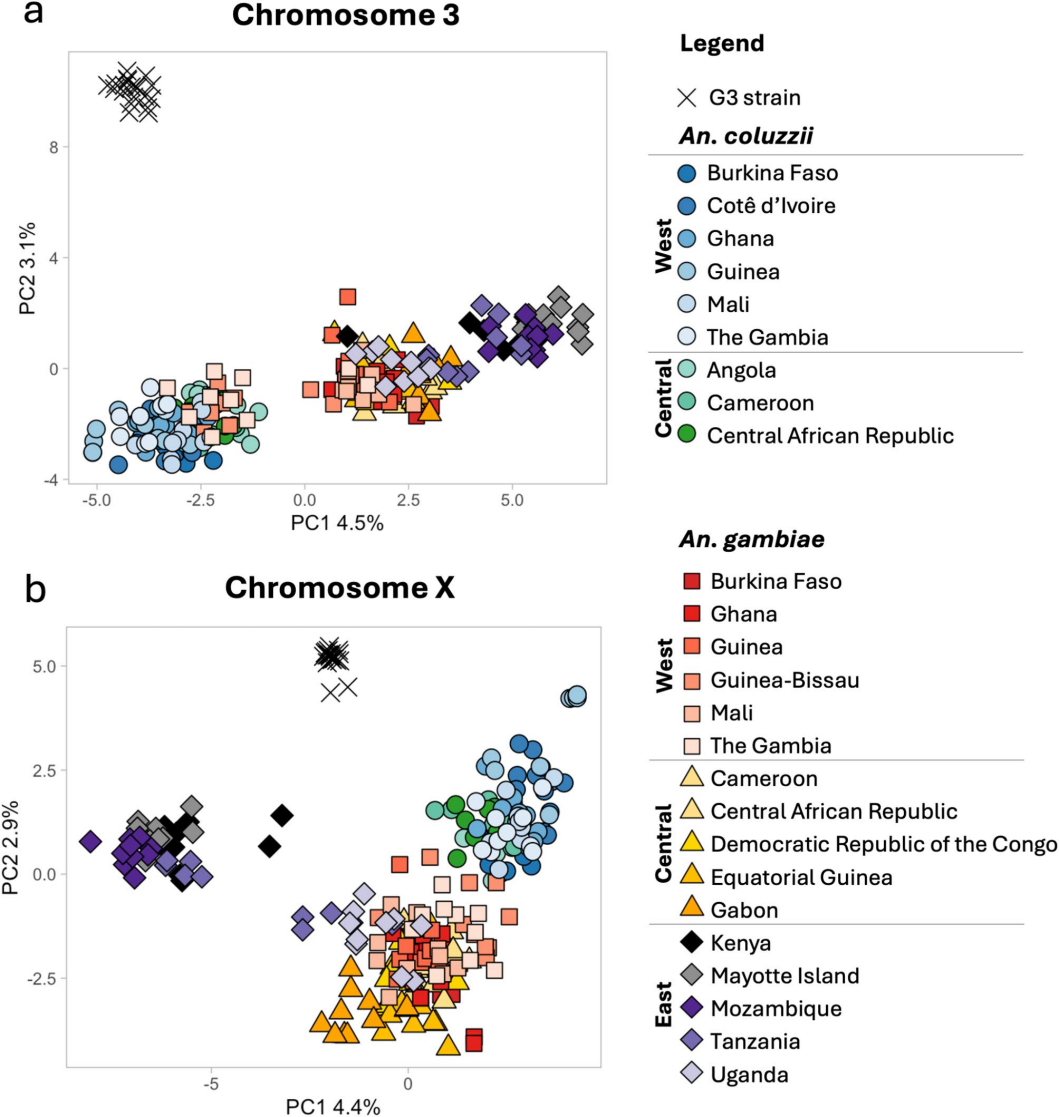
Principal component analysis (PCA). Plot of the first two components of a PCA depicting relationships among populations of the G3 strain and natural populations of *An. gambiae* and *An. coluzzii*. Analyses were based on a random subset of 5000 high-quality biallelic SNPs on chromosome 3 (**a**), and the X chromosome (**b**).

A random subset of 5000 high-quality biallelic SNPs was selected from the genome sequences of individual field-collected mosquitoes from 18 sites across sub-Saharan Africa (Fig. 1). Principal Component Analyses (PCA) were conducted using these SNPs, as well as a sample of the G3 strain (Fig. 2). SNPs on chromosome 2 were excluded to avoid a potentially confounding influence due to the numerous paracentric inversions present on this chromosome. Results distinguished the G3 strain specimens from all natural populations of *An. gambiae* and *An. coluzzii* across Africa. Four distinct clusters were identified: the G3 strain; *An. coluzzii* from Western and Central African populations; *An. gambiae* from West and Central Africa; and *An. gambiae* from East Africa.

**Fig. 3:**
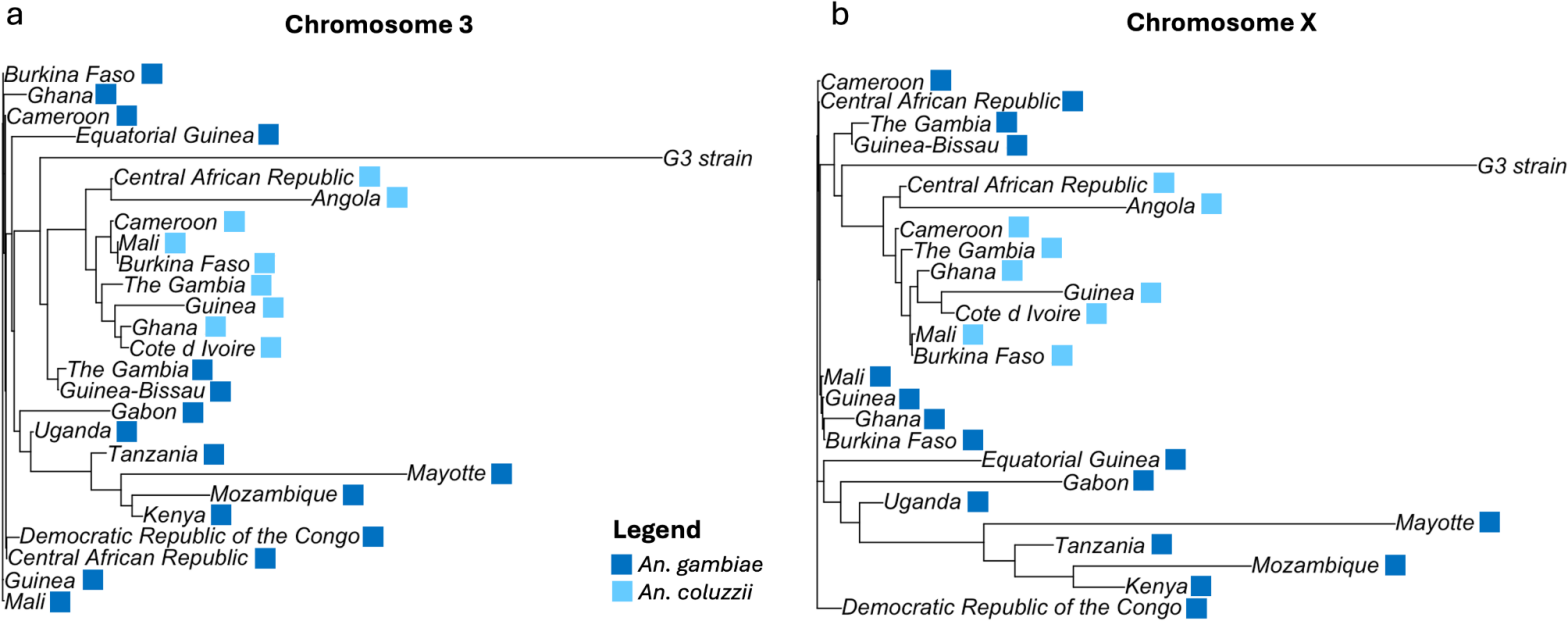
*F*_*ST*_ analyses. Neighbor-joining tree based on pairwise *F*_*ST*_ values between the G3 strain and natural populations of *An. gambiae* (dark blue) and *An. coluzzii* (light blue). The analysis was performed with SNPs on chromosome 3 (**a**) and the X chromosome (**b**).

Pairwise *F*_*ST*_ calculations were used to establish relationships among natural populations of *An*. *coluzzii* and *An. gambiae* and to explore their relationship to the G3 strain (Tables S1, S2). To facilitate visualization of these relationships, *F*_*ST*_ values were used to construct an unrooted phylogenetic tree using the neighbor-joining (NJ) algorithm (Fig. 3). Several population groups are apparent in the trees. *An. coluzzii* formed a distinct group that included population samples over most of its range (West/Central sub-Saharan Africa). The genetic structure of *An. gambiae* was more complex consisting of a group of closely related West African populations and an eastern group of more highly diverged populations. Within the East African group, a population on the island of Mayotte is the most highly diverged. As expected, the G3 strain was the most highly diverged of all populations. The G3 population’s position on the NJ tree reflects the fact that it is a hybrid population of *An. gambiae* and *An. coluzzii*.

### Statistical metrics

**Fig. 4:**
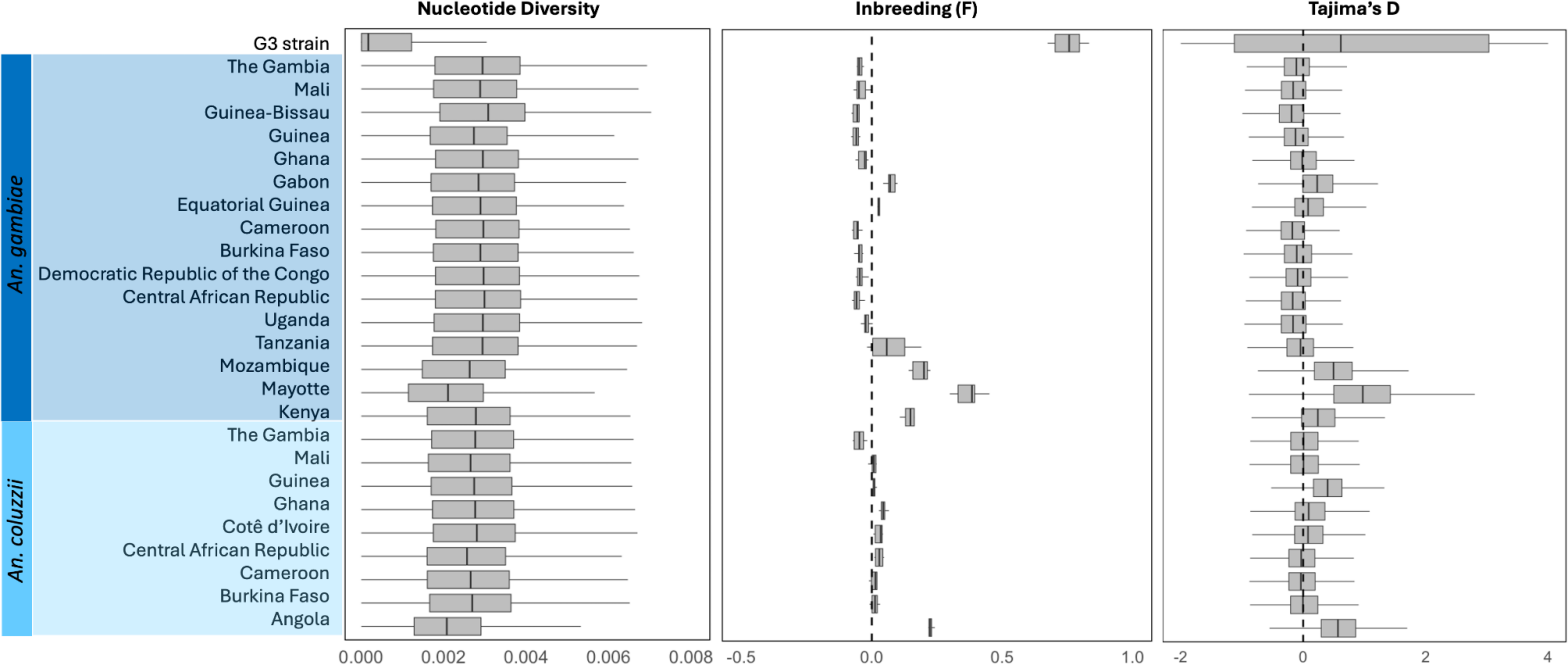
Population genetic statistics. Metrics are grouped by sampling locations and species. Nucleotide diversity and Tajima’s D were calculated for non-overlapping windows of 20 kb. Inbreeding was calculated using variants across the whole genome. The midline in all boxplots represents the median, with the upper (75th percentile) and lower (25th percentile) limits, whiskers show maximum and minimum values, and outliers are not shown.

Three statistical metrics were calculated for the G3 strain and for samples of natural populations of *An. gambiae* and *An. coluzzii* (Fig. 4). The G3 strain exhibited characteristics typical of a long-established laboratory colony, which markedly distinguishes them from natural populations. The G3 strain had the lowest nucleotide diversity, and the highest inbreeding coefficient. G3 also had the largest range of Tajima’s D values with a positive mean, indicative of population contraction; typical for a population experiencing a founder effect as expected for a laboratory colony population. A similar trend was observed in the population of *An. gambiae* from Mayotte Island and, to a lesser extent, *An. coluzzii* from Angola.

### Divergence genome scan

**Fig. 5:**
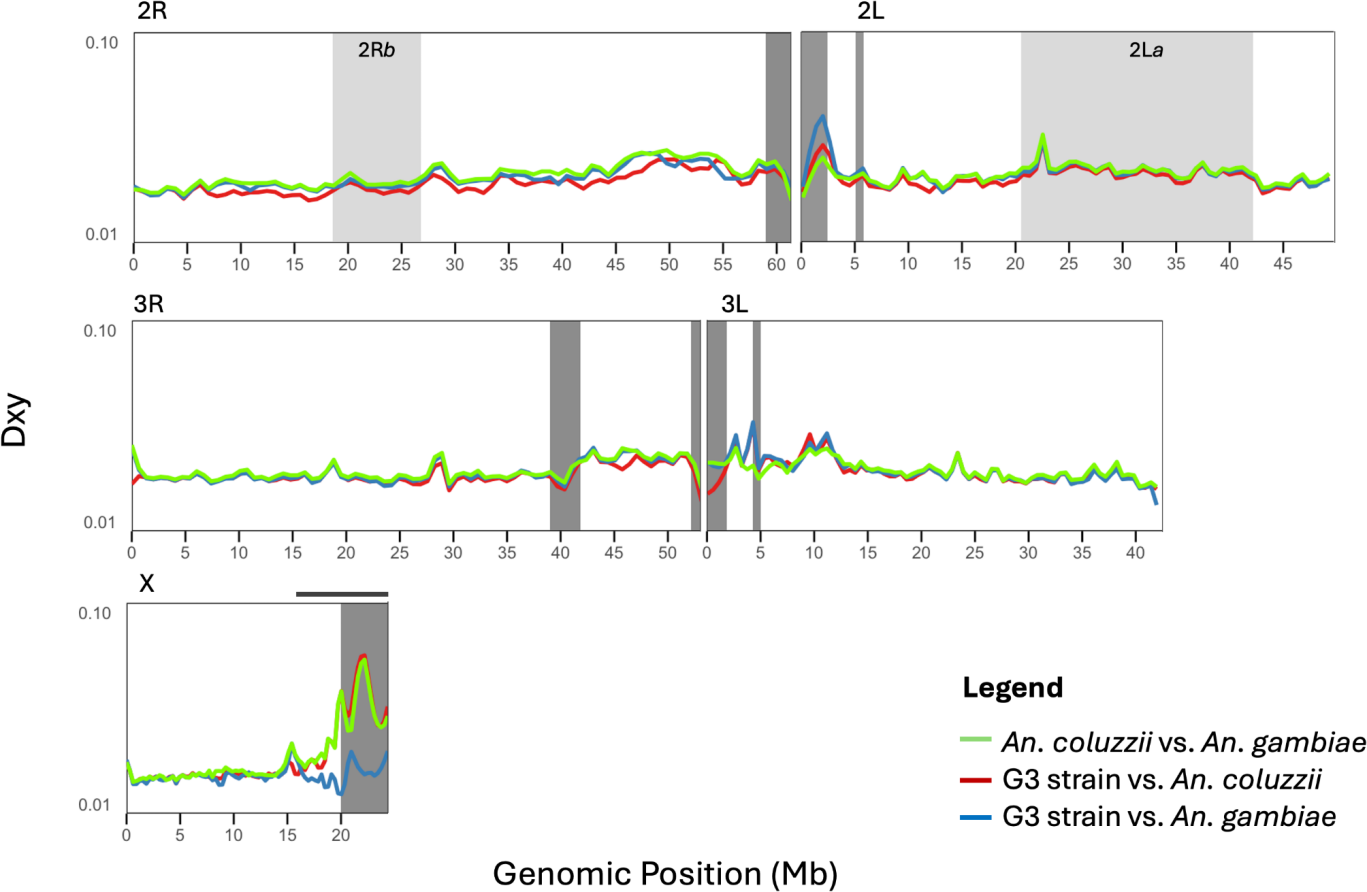
Genome-wide windowed divergence. Comparisons between the G3 strain and natural populations of both *An. gambiae* and *An. coluzzii*. Genetic divergence was calculated in 50 kb windows across the genome. Light gray shaded regions highlight the location of two paracentric inversions on chromosome 2, dark shaded regions denote heterochromatin, and the dark bar on the X chromosome highlights the limits of the ‘island of speciation’ as consistently defined in previous studies.

A windowed-Dxy analysis was used to describe genome-wide divergence between the G3 strain, field-collected *An. gambiae*, and *An. coluzzii*. In general, high divergence was detected at pericentromeric regions of the genomes, known to be regions at which *An. gambiae* and *An. coluzzii* are highly diverged (Fig. 5). The G3 strain showed higher divergence from *An. gambiae* on the autosomes. Conversely, in the pericentromeric region of the X chromosome, the G3 strain exhibited higher divergence from *An. coluzzii* (Fig. 5).

### Species-diagnostic markers

**Fig. 6:**
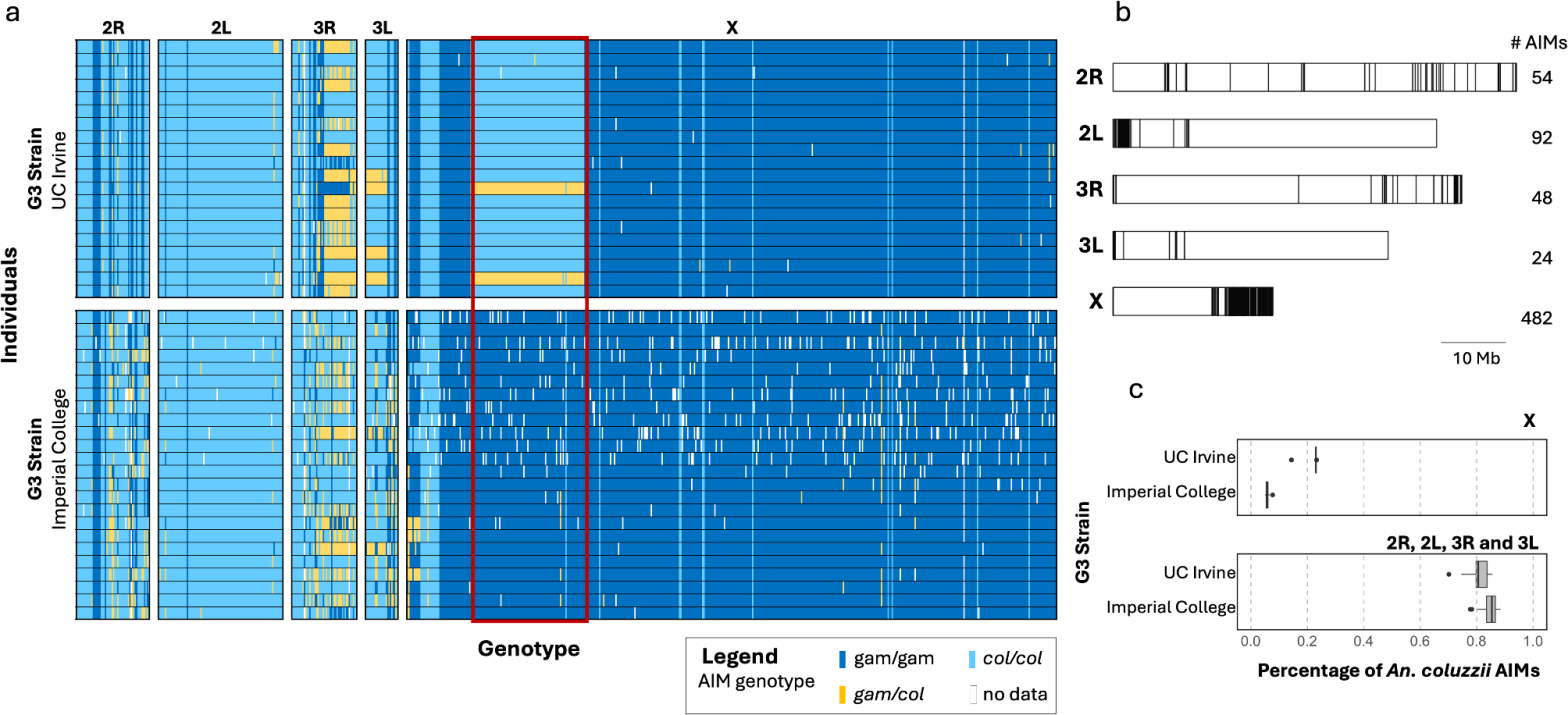
Ancestry informative markers (AIM) genotypes. A set of 700 SNPs from Ag1000G (phase 2) analysis, where one allele is at or near fixation in one species, and an alternative allele in the other. (**a**) Homozygous genotypes for the *An. gambiae* allele are shown in dark blue, homozygous for *An. coluzzii* are in light blue, and heterozygotes are represented in yellow. Each row represents an individual mosquito, grouped by the facility where the G3 strain was maintained. SNPs are grouped by linkage association on chromosomes 2R, 2 L, 3R, 3 L and X. Red box highlights divergence region on X chromosome between G3 strains: from UC Irvine and Imperial College. (**b**) Location of each AIM and number of markers across the genome. (**c**) Percentage of *An. coluzzii* AIMs on X-chromosome and autosomes for each G3 strain. **Declarations**.

We investigated a set of 700 ancestry-informative markers (AIMs) used by the *Anopheles gambiae* 1000 Genomes consortium (Ag1000G) phase 2^44^ to distinguish *An. coluzzii* from *An. gambiae* (Table S3). The G3 strain revealed a complex mosaic genome structure with ancestry contributions from both *An. coluzzii* and *An. gambiae*. Specifically, the G3 strain exhibited primarily *An. coluzzii* ancestry on autosomes, while most AIMs on the X chromosome were of *An. gambiae* ancestry (Fig. 6). We obtained AIMs for a second G3 sub-strain, maintained at Imperial College^45^, and compared them with the G3 strain in this study (University of California, Irvine). This comparison revealed a considerable difference in the X chromosome, with a roughly 4 Mbp region of the X chromosome distinguishing the two sub-strains (Fig. 6). Additionally, the G3 strain differs on AIM patterns from hybrids or intermediates between *An. gambiae* and *An. coluzzii* found in nature (Supplementary Fig. 1).

## Discussion

The G3 strain has been a cornerstone in the study of malaria vector biology. Although it has been long known that G3 is an interspecific hybrid strain, until this report, a careful analysis at the genome level has not been conducted. This information relates to the validity of extrapolating research results obtained in the laboratory using the G3 strain to mimic natural populations of these mosquito species and may explain those cases in which these results are discordant. Our finding that, even where G3 sub-colonies are maintained in different laboratories, significant differences may arise (either through genetic drift or inadvertent contamination but other strains) and these differences may explain cases in which results between labs cannot be replicated.

The origin of the G3 strain dates to 1975 from individuals collected in The Gambia where both *An. coluzzii* and *An. gambiae* occur in sympatry^27^. In 1975 these two species were not distinguished taxonomically, with the taxon *An. coluzzii* subsumed under the designation *Anopheles gambiae*. It is likely that the G3 colony established at this time included a mixture of both *An. coluzzii* and *An. gambiae* individuals and possibly included some naturally occurring hybrids. These two species rarely hybridize in nature and hybrids that are produced have reduced fitness^46^; however, in laboratory settings they readily hybridize and produce hybrid individuals that experience no apparent reduction in fitness^47^. This scenario likely produced the extant G3 strain.

In analysis of genetic similarity (Figs. 2 and 3) the G3 strain was not grouped with either of the two species from which it is derived. The G3 strain appears to represent a discrete and unique taxonomic unit not found in nature. Comparative analyses employing conventional population genetic descriptors for the G3 strain and conspecific natural populations (Fig. 4) met expectations for a typical insect laboratory colony and are concordant with earlier studies of the species described here^33^. These expectations include low nucleotide diversity and heterozygosity and high inbreeding, likely resulting from population bottlenecks at the time this colony was established, and sporadic population crashes typically experienced during long-term mosquito colony maintenance. These results reinforce concerns about assuming that experimental results based on laboratory colonies apply to individuals in natural populations^44^.

Examination of the G3 genome included a sliding window analysis comparing the G3, *An. coluzzii*, and *An. gambiae* genomes (Fig. 5). Genetic sequence divergence (D_XY_) was used to describe relationships across the genome. The G3 strain was most similar to *An. coluzzii* across chromosome 2 and on chromosomes 3R from approximately 25 Mb extending to 20 Mb on 3 L (Fig. 5). On the X chromosome, G3 was more similar to *An. gambiae*. These results were consistent with genotype analysis of 700 ancestry informative markers (AIMS) illustrated in Fig. 6a. The AIMs were not evenly distributed across the genome with 69% on the X chromosome (Fig. 6b). The genomes of *An. coluzzii* and *An. gambiae* differ by only an estimated 2% with divergence largely contained within a few regions of the genome referred to as “islands of speciation”^46^. The X chromosomes are known to include a 4 Mbp island of speciation thought to be associated with assortative mating^47^. Surprisingly, the AIM genotype analysis revealed significant differentiation between two G3 sub-colonies, one maintained at the University of California, Irvine and the second at Imperial College, London. The major difference between these two sub-colonies is a 0.62 Mbp region of the X chromosome containing 84 AIMs. In the Imperial strain this region includes all *An. gambiae* associated AIMs, whereas in the UC Irvine strain these AIMs are all *An*. *coluzzii*. It is unclear what may have given rise to these differences or if it may be associated with any differences in phenotype, although this region of the genome is thought to be involved with mating.

In conclusion, it should be recognized that the G3 strain has a unique mosaic genome. Its genome consists of two autosomes of mostly *An. coluzzii* ancestry and an X chromosome of *An. gambiae* origin. The G3 strain should not be referred to as *Anopheles gambiae* nor *Anopheles coluzzii*. Laboratory strains of both “pure” *An. gambiae s.s*. and *An. coluzzii* are available from the Malaria Research and Reference Reagent Resource Center (BEI Resources). Given the wide variety of well-characterized strains available to researchers, the choice of strain should be carefully determined based on the specific needs of the study. In the case of G3, the source is particularly important, as variations between institutes may impact result interpretation. For research intended to have direct field applicability, it may also be beneficial to establish new colonies or periodically refresh existing ones with field-caught mosquitoes.

## Methods

### Sampling and sequencing

In this paper we combined publicly available whole genome sequence data with newly generated sequence data from the G3 (Malaria Research and Reference Reagent Resource Center, Atlanta, USA; stock number MRA112) sub-colony maintained in insectary facilities at the Department of Molecular Biology and Biochemistry, School of Biological Sciences, University of California, Irvine (UCI). The colony was established in 2016 from approximately 100 individuals provided by MR4 and was maintained until the receipt of the samples in August 2022. According to direct communication with Dr. Anthony James, the colony is housed in cages containing no more than 200–300 mosquitoes each, with the total number of cages fluctuating depending on ongoing experimental needs. For the newly sequenced samples, DNA was extracted from 20 individual whole adult females using a Qiagen BioSprint robot and published methods^48^. DNA was quantified by fluorometry with a Qubit dsDNA HS Assay kit (Thermo Fisher Scientific, Inc.) and 10 ng of DNA was used to prepare a barcoded sequencing library for each individual using the KAPA HyperPlus kit (Roche, Inc.) following the manufacturer’s instructions. Libraries were pooled, and PE150 sequenced on a HiSeq 3000/4000 instrument (Illumina, Inc.).

The publicly available data used in this study included: (1) whole genome sequences of 12 male and 12 female G3 strain pupae maintained at the Department of Life Sciences, Imperial College London, UK, downloaded as raw PE100 Illumina reads from the Sequence Read Archive; (2) whole genome sequences of wild-caught individuals of *An. gambiae* (*N* = 160) and *An. coluzzii* (*N* = 90) from 18 countries, collected over 22 years (1993–2015) (Fig. 1; Supplementary Table S4); and (3) hybrids or intermediate between *An. gambiae* and *An. coluzzii* from 5 countries (Supplementary Table S4). Sequences from 2 to 3 were downloaded as VCF files from MalariaGEN, representing a subset of 10 specimens per country from The *Anopheles gambiae* 1000 Genomes Consortium phase 3 dataset^49^.

### Sequencing data processing

The raw reads from UCI and Imperial College were quality trimmed and adapters removed using *Trimmomatic* v0.36^50^ and the appropriate adapter file for each dataset (Supplementary Information). Orphan reads were discarded. Paired reads were aligned to the AgamP4^51^ genome with BWA-MEM v0.7.17^52^ and data from multiple sequencing runs per sample were merged as necessary with Sambamba^53^. Resulting SAM files were modified with *fixmate* then sorted and converted to BAM using SAMtools^54^. BAM files were modified with *picardtools*^55^, SetNmMdAndUqTags and duplicates were marked with bammarkduplicates. Indel realignment was performed using GATK version 3.7-0^56^ RealignerTargetCreator and IndelRealigner. Variants were called using GATK version 3.7-0 UnifiedGenotyper with output mode EMIT_ALL_SITES, genotyping_mode GENOTYPE_GIVEN_ALLELES, and supplied with a VCF file containing the alleles used by Ag1000G at each genomic site. Thus, the resulting genotype calls utilized the same ordered set of ALT alleles at each site as the Ag1000G dataset.

### DNA sequence data merging and filtering

Genotype call data for all Ag1000G samples of interest were downloaded as individual VCF files from MalariaGEN and were merged with VCF files from G3 samples using BCFtools^57^. For all analyses except AIMs marker analysis, data was filtered to retain only bi-allelic SNPs in the accessible genome (based on accessibility map from Ag1000G) with no more than 5% missing data.

### Population genomics analysis

Additional SNP filtering was performed for population structure analysis using Principal Component Analysis (PCA) and pairwise *F*_*ST*_. The dataset was filtered to exclude minor allele frequency (MAF) < 1%. Both analyses were performed based on SNPs on chromosome 3 and chromosome X only. Chromosome 2 was omitted to avoid the paracentric inversions common in this part of the genome^58^. Heterochromatin regions on chromosome 3R and 3 L were also filtered out^51^. PCA was performed after pruning for linkage disequilibrium (LD) using *scikit-allel* v1.2.0^59^. Hudson’s estimator was used for pairwise fixation index *F*_*ST*_ calculations, implemented in *scikit-allel*. Neighbor-joining tree and plots were created in R using the *ggplot2* and *ggtree* packages. VCFtools v.01.16^60^ was used to calculated nucleotide diversity (π) Tajima’s D in nonoverlapping windows of 20 kb. Inbreeding was also calculated in VCFtools using the method-of-moments approach.

### Ancestry informative markers

Filtering with BCFtools^57^ was used to collect genotypes of all G3 samples at SNPs determined to be Ancestry Informative Markers (AIMs) by the Ag1000G. These included 700 SNPs fixed for distinct alleles between *An. gambiae* and *An. coluzzii* (Supplementary Table 3).

### Genome scan

For windowed calculation of divergence between two populations (Dxy), the filtered SNP genotype data described above was further filtered and converted to DNA letter codes using the *parseVCF.py* program from the Simon Martin laboratory (https://simonmartinlab.org). A window size of 50 kb and minimum of 500 SNPs was used. Resulting data were plotted in R.

## Electronic supplementary material

Below is the link to the electronic supplementary material.

Supplementary Material 1

Supplementary Material 2

## Data Availability

New whole genome sequence data included in this study is deposited in NCBI GenBank under BioProject ID PRJNA1163388.
