# Supplementary material for: Defining the genetics of the widely used G3 strain of the mosquito, Anopheles gambiae
Source: Sci Rep. 2025 Apr 16;15:13142. doi: 10.1038/s41598-025-96391-y (PMC12003814; doi:10.1038/s41598-025-96391-y)
Supplement: Supplementary file 1 — Supplementary Material 1 [file 41598_2025_96391_MOESM1_ESM.pdf]

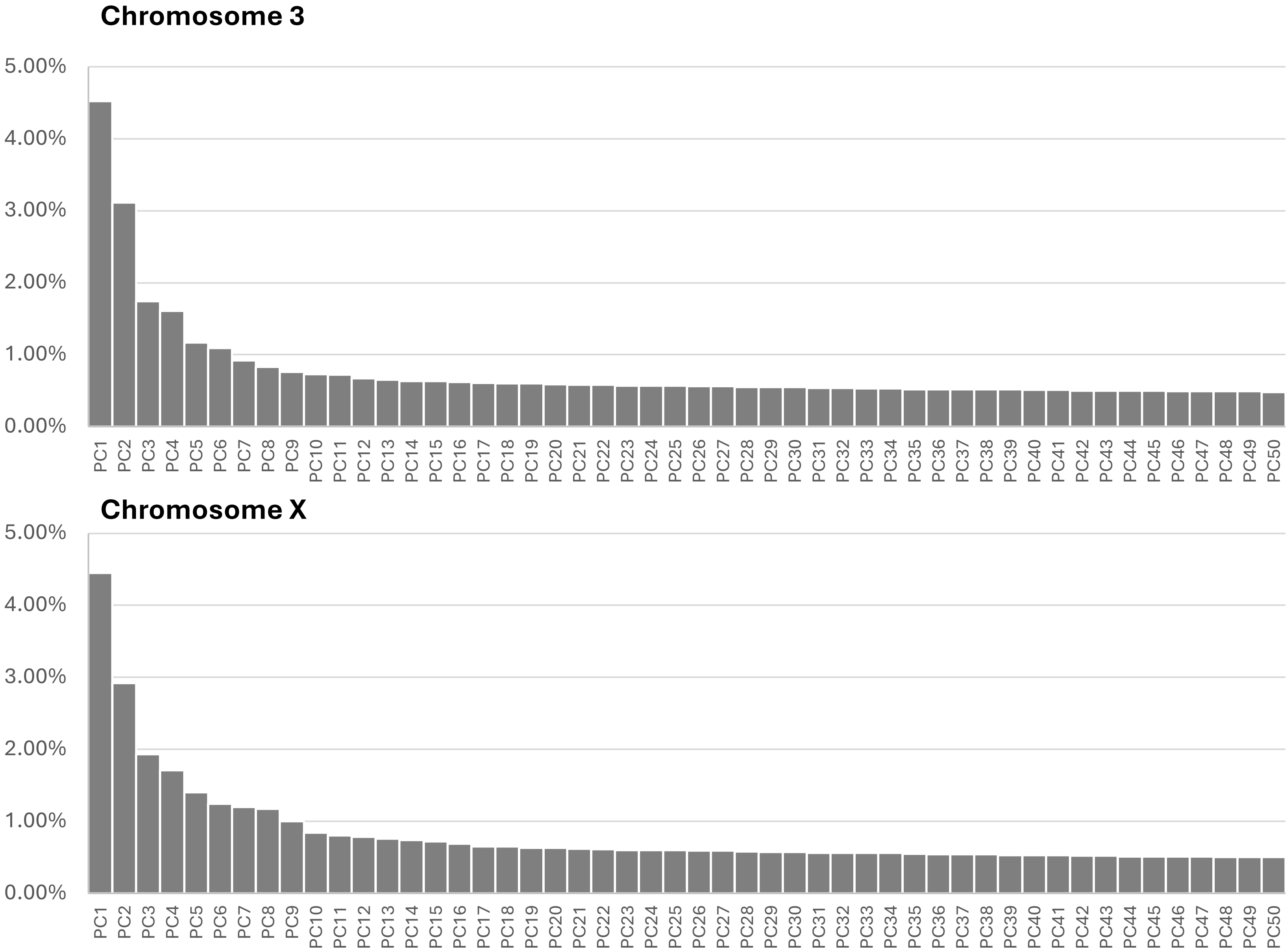

Supplementary Figure 1. Scree plot for principal component analysis. Scree plots corresponding to the PCAs shown in Figure 2. The x-axis represents the first 50 PCs, while y-axis indicates the proportion of variance explained by each component.

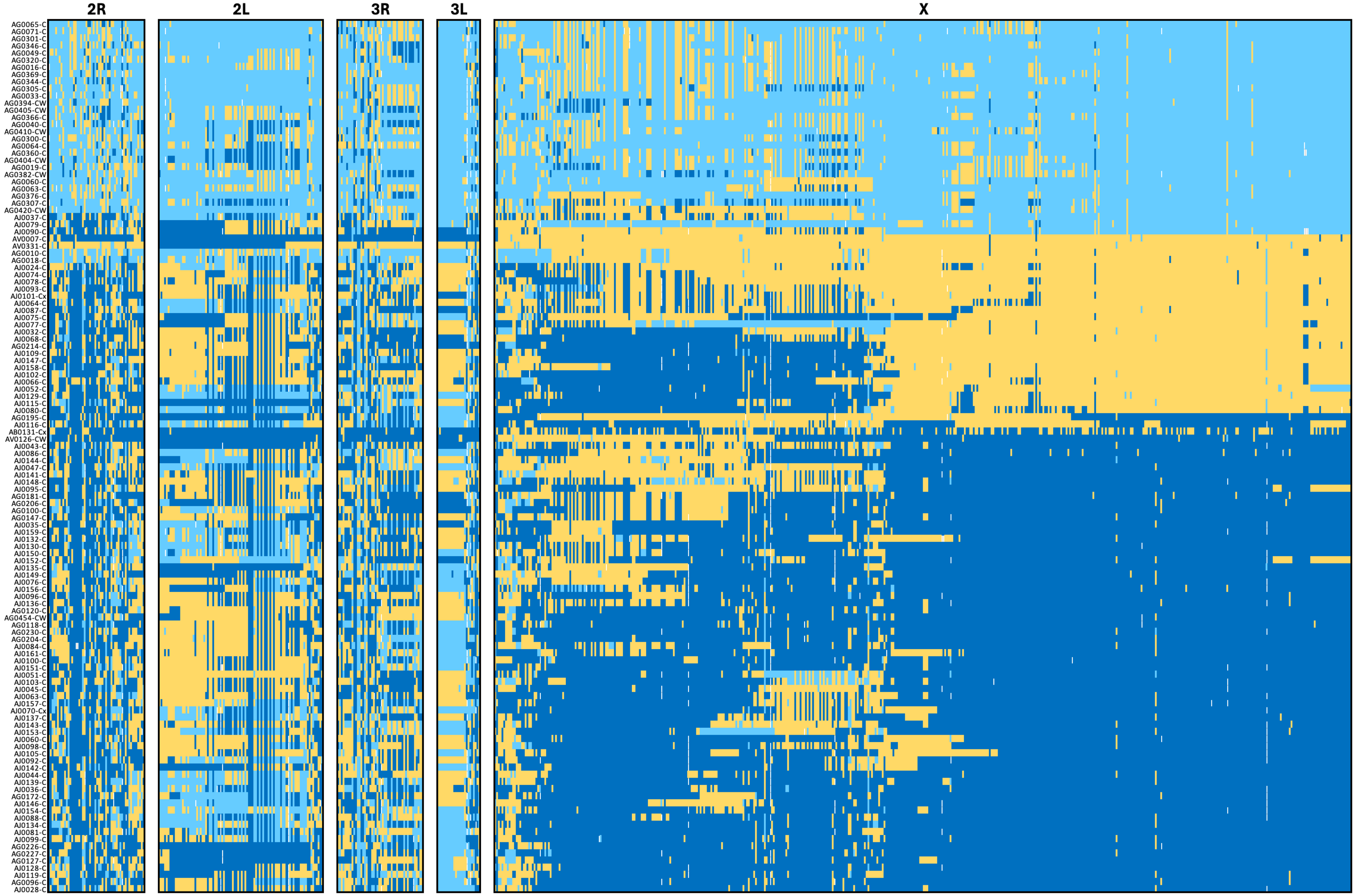

Legend

AIM genotypes

gam/gam *col/col*
gam/col no data

**Country**	***N***	**SampleID**
Burkina Faso	1	AB0131-Cx
Guinea	2	AV0007-C, AV0126-CW
Guinea-Bissau	73	AJ0###-C
Mali	1	AV0331-C
The Gambia	45	AG0###-C

Supplementary Figure 2. Ancestry informative markers (AIM) genotypes. A set of 700 SNPs from Ag1000G (phase 2) analysis between *An. gambiae* and *An. coluzzii*, where one allele is at or near fixation in one species, and an alternative allele in the other. This set of 122 samples was field-collected in 5 countries in West Africa: Burkina Faso (*N*=1), Guinea (*N*=2), Guinea-Bissau (*N*=73), Mali (*N*=1), The Gambia (*N*=45). They were defined as intermediate between the two species by Ag1000G.

Supplementary Table S1 - Pairwise FSTs using SNPs on chromosome 3 between G3 strain and natural populations of An. gambiae and An. coluzzii across Africa.

	G3_UCMI	Angola_col	Burkina_col	Burkina_gam	Cameroon_c	Cameroon_g	Central_col	Central_gam	Congo_gam	Cote_col	Equatorial_g	Gabon_gam	Ghana_col	Ghana_gam	Guinea_col	Guinea_gam	Guinea-Biss	Kenya_gam	Mali_col	Mali_gam	Mayotte_gan	Mozambique	Tanzania_ga	TheGambia_	TheGambia_	Uganda_gam
**G3_UCMI**		0.40837767	0.3021154	0.29384688	0.29846146	0.29268633	0.30229179	0.29301363	0.29761178	0.30345032	0.32093731	0.33366674	0.30368887	0.30363727	0.33319579	0.29467379	0.28890658	0.32245756	0.30173746	0.29255999	0.47216464	0.36915467	0.30600549	0.30201869	0.29238319	0.29394721
**Angola_col**	0.40837767		0.118727	0.13695378	0.09807373	0.13416211	0.08849115	0.13509817	0.13979431	0.12388452	0.15135418	0.16159316	0.12356587	0.14312156	0.15189649	0.13700408	0.1312234	0.17806011	0.11836258	0.13426153	0.29009212	0.20770446	0.15187537	0.13191695	0.13385743	0.13740183
**Burkina_col**	0.3021154	0.118727		0.05390179	0.0019888	0.05303419	0.02033185	0.05386036	0.05875297	0.01556929	0.07600078	0.08926807	0.01605907	0.06005541	0.04537187	0.05318906	0.03567069	0.10051152	-0.0004266	0.05026106	0.21636976	0.13206714	0.07494015	0.02436425	0.0380592	0.05697604
**Burkina_gam**	0.29384688	0.13695378	0.05390179		0.04980972	0.00057014	0.05121923	0.00188568	0.00725776	0.05989908	0.03252154	0.04904512	0.06134875	0.01045425	0.08482736	0.00047108	0.02293337	0.06827589	0.05355442	0.00091834	0.18527572	0.09680024	0.03316518	0.05769172	0.02635367	0.01099825
**Cameroon_col**	0.29846146	0.09807373	0.0019888	0.04980972		0.04869008	0.01194164	0.04976207	0.05446087	0.01520897	0.07066446	0.08357443	0.01482109	0.05630917	0.04473528	0.04926142	0.03350557	0.09603639	0.00206136	0.04654361	0.21126305	0.12773975	0.07027818	0.02349193	0.0359262	0.05266303
**Cameroon_gam**	0.29268633	0.13416211	0.05303419	0.00057014	0.04869008		0.04933506	0.00071734	0.00660597	0.05865419	0.03112487	0.04386026	0.06022065	0.01073783	0.08358449	0.00169951	0.02301765	0.06444585	0.05245095	0.00183011	0.18218469	0.0930802	0.03015834	0.05671016	0.02627527	0.00919441
**Central_col**	0.30229179	0.08849115	0.02033185	0.05121923	0.01194164	0.04933506		0.05015782	0.05510416	0.02303532	0.07042533	0.08340779	0.02230236	0.05754631	0.05233275	0.05121129	0.0381612	0.09604113	0.0200273	0.04819242	0.21097679	0.12733522	0.07053988	0.03254289	0.04064432	0.05269248
**Central_gam**	0.29301363	0.13509817	0.05386036	0.00188568	0.04976207	0.00071734	0.05015782		0.00576995	0.05991377	0.0317493	0.04720003	0.06135927	0.0112826	0.08425881	0.00222197	0.02294054	0.06515963	0.05356923	0.00210548	0.18298769	0.09400329	0.03027925	0.05744997	0.02605803	0.00832104
**Congo_gam**	0.29761178	0.13979431	0.05875297	0.00725776	0.05446087	0.00660597	0.05510416	0.00576995		0.06457487	0.03670517	0.051671	0.06604592	0.01696045	0.08894248	0.00758222	0.02820208	0.06809583	0.05811054	0.00796829	0.18630747	0.09689311	0.03299675	0.06207099	0.03158838	0.01180569
**Cote_col**	0.30345032	0.12388452	0.01556929	0.05989908	0.01520897	0.05865419	0.02303532	0.05991377	0.06457487		0.08139368	0.09479484	0.00769625	0.06594381	0.03519698	0.05955017	0.0365501	0.10580523	0.01593181	0.05666685	0.22036096	0.13766934	0.08095769	0.02411049	0.03964654	0.06308657
**Equatorial_gam**	0.32093731	0.15135418	0.07600078	0.03252154	0.07066446	0.03112487	0.07042533	0.0317493	0.03670517	0.08139368		0.06929244	0.08246109	0.04179732	0.10560208	0.03268219	0.04946074	0.08820286	0.07553257	0.03232471	0.20393687	0.11571304	0.0554185	0.07979179	0.05303081	0.03706262
**Gabon_gam**	0.33366674	0.16159316	0.08926807	0.04904512	0.08357443	0.04386026	0.08340779	0.04720003	0.051671	0.09479484	0.06929244		0.09550244	0.05821512	0.11859183	0.04895881	0.06531636	0.08996946	0.08882132	0.04909585	0.20491619	0.11677465	0.05971308	0.09348339	0.06839804	0.04982915
**Ghana_col**	0.30368887	0.12356587	0.01605907	0.06134875	0.01482109	0.06022065	0.02230236	0.06135927	0.06604592	0.00769625	0.08246109	0.09550244		0.06700764	0.03936246	0.06061154	0.03787601	0.10674304	0.01674697	0.05821822	0.2214939	0.13857957	0.08201456	0.02436569	0.0407857	0.0646021
**Ghana_gam**	0.30363727	0.14312156	0.06005541	0.01045425	0.05630917	0.01073783	0.05754631	0.0112826	0.01696045	0.06594381	0.04179732	0.05821512	0.06700764		0.09031484	0.01066391	0.0311348	0.07644179	0.05966639	0.01117491	0.19359021	0.10513103	0.04217541	0.06403658	0.03475348	0.02088676
**Guinea_col**	0.33319579	0.15189649	0.04537187	0.08482736	0.04473528	0.08358449	0.05233275	0.08425881	0.08894248	0.03519698	0.10560208	0.11859183	0.03936246	0.09031484		0.08368738	0.05663364	0.13000333	0.04528111	0.08141688	0.24318853	0.16121684	0.10465844	0.04304127	0.06003948	0.08749209
**Guinea_gam**	0.29467379	0.13700408	0.05318906	0.00047108	0.04926142	0.00169951	0.05121129	0.00222197	0.00758222	0.05955017	0.03268219	0.04895881	0.06061154	0.01066391	0.08368738		0.02259395	0.06845504	0.05284114	0.00091022	0.18577823	0.09696798	0.03327263	0.05733946	0.02554323	0.01162723
**Guinea-Bissau_gam**	0.28890658	0.1312234	0.03567069	0.02293337	0.03350557	0.02301765	0.0381612	0.02294054	0.02820208	0.0365501	0.04946074	0.06531636	0.03787601	0.0311348	0.05663364	0.02259395		0.08053517	0.03545564	0.02163026	0.19640437	0.11066285	0.04963105	0.01744354	0.00386258	0.02924044
**Kenya_gam**	0.32245756	0.17806011	0.10051152	0.06827589	0.09603639	0.06444585	0.09604113	0.06515963	0.06809583	0.10580523	0.08820286	0.08996946	0.10674304	0.07644179	0.13000333	0.06845504	0.08053517		0.09999472	0.06740401	0.14314821	0.03918831	0.0115771	0.10509416	0.08385852	0.05101224
**Mali_col**	0.30173746	0.11836258	-0.0004266	0.05355442	0.00206136	0.05245095	0.0200273	0.05356923	0.05811054	0.01593181	0.07553257	0.08882132	0.01674697	0.05966639	0.04528111	0.05284114	0.03545564	0.09999472		0.04991013	0.21579296	0.13156064	0.07438062	0.02367114	0.03755963	0.0565629
**Mali_gam**	0.29255999	0.13426153	0.05026106	0.00091834	0.04654361	0.00183011	0.04819242	0.00210548	0.00796829	0.05666685	0.03232471	0.04909585	0.05821822	0.01117491	0.08141688	0.00091022	0.02163026	0.06740401	0.04991013		0.18453995	0.0963021	0.03291583	0.05474146	0.02506681	0.01060844
**Mayotte_gam**	0.47216464	0.29009212	0.21636976	0.18527572	0.21126305	0.18218469	0.21097679	0.18298769	0.18630747	0.22036096	0.20393687	0.20491619	0.2214939	0.19359021	0.24318853	0.18577823	0.19640437	0.14314821	0.21579296	0.18453995		0.16026338	0.13927953	0.22022189	0.19942675	0.16943449
**Mozambique_gam**	0.36915467	0.20770446	0.13206714	0.09680024	0.12773975	0.0930802	0.12733522	0.09400329	0.09689311	0.13766934	0.11571304	0.11677465	0.13857957	0.10513103	0.16121684	0.09696798	0.11066285	0.03918831	0.13156064	0.0963021	0.16026338		0.03964053	0.13685187	0.11400418	0.0784109
**Tanzania_gam**	0.30600549	0.15187537	0.07494015	0.03316518	0.07027818	0.03015834	0.07053988	0.03027925	0.03299675	0.08095769	0.0554185	0.05971308	0.08201456	0.04217541	0.10465844	0.03327263	0.04963105	0.0115771	0.07438062	0.03291583	0.13927953	0.03964053		0.0789927	0.05315172	0.01699336
**TheGambia_col**	0.30201869	0.13191695	0.02436425	0.05769172	0.02349193	0.05671016	0.03254289	0.05744997	0.06207099	0.02411049	0.07979179	0.09348339	0.02436569	0.06403658	0.04304127	0.05733946	0.01744354	0.10509416	0.02367114	0.05474146	0.22022189	0.13685187	0.0789927		0.02187181	0.06092593
**TheGambia_gam**	0.29238319	0.13385743	0.0380592	0.02635367	0.0359262	0.02627527	0.04064432	0.02605803	0.03158838	0.03964654	0.05303081	0.06839804	0.0407857	0.03475348	0.06003948	0.02554323	0.00386258	0.08385852	0.03755963	0.02506681	0.19942675	0.11400418	0.05315172	0.02187181		0.03278223
**Uganda_gam**	0.29394721	0.13740183	0.05697604	0.01099825	0.05266303	0.00919441	0.05269248	0.00832104	0.01180569	0.06308657	0.03706262	0.04982915	0.0646021	0.02088676	0.08749209	0.01162723	0.02924044	0.05101224	0.0565629	0.01060844	0.16943449	0.0784109	0.01699336	0.06092593	0.03278223	

Supplementary Table S2 - Pairwise F_{ST}s using SNPs on chromosome X between G3 strain and natural populations of *An. gambiae* and *An. coluzzii* across Africa.

	G3_UCMI	Angola_col	Burkina_col	Burkina_gam	Cameroon_c	Cameroon_g	Central_col	Central_gam	Congo_gam	Cote_col	Equatorial_g	Gabon_gam	Ghana_col	Ghana_gam	Guinea_col	Guinea_gam	Guinea-Biss	Kenya_gam	Mali_col	Mali_gam	Mayotte_gan	Mozambique	Tanzania_ga	TheGambia_	TheGambia_	Uganda_gam
**G3_UCMI**		0.44405322	0.30356816	0.29270059	0.29852414	0.29018009	0.30700486	0.2920514	0.30395078	0.32408728	0.3840922	0.41923653	0.31074863	0.30904664	0.3827524	0.29374503	0.29547895	0.43175084	0.30635301	0.29018396	0.63378162	0.52255369	0.34599189	0.30477741	0.30599453	0.29181963
**Angola_col**	0.44405322		0.1356892	0.13650623	0.10843813	0.13328601	0.10426853	0.13628901	0.1433981	0.15351862	0.20057307	0.2359484	0.14161122	0.14960597	0.20363615	0.1359417	0.14284468	0.26883025	0.1366132	0.13412442	0.38354765	0.32197863	0.20070187	0.1402187	0.15177958	0.14290916
**Burkina_col**	0.30356816	0.1356892		0.04667293	0.00712973	0.04420849	0.02238547	0.04627125	0.05616128	0.01593056	0.11454943	0.14963107	0.01208154	0.05971987	0.0682156	0.04792991	0.03751076	0.17297418	0.001737	0.04366511	0.28636356	0.22674105	0.10862156	0.01063285	0.0494066	0.05328676
**Burkina_gam**	0.29270059	0.13650623	0.04667293		0.04084802	0.0020872	0.04467515	0.00190123	0.01301965	0.066866	0.07597208	0.113655	0.05546627	0.01303225	0.11389228	-3.97E-05	0.01742766	0.14494354	0.0483443	-0.0003485	0.26246899	0.19923801	0.07362196	0.04716338	0.0235359	0.01141785
**Cameroon_c**	0.29852414	0.10843813	0.00712973	0.04084802		0.03780248	0.00922667	0.03993437	0.04915211	0.02659763	0.10846292	0.14395158	0.01310692	0.05409953	0.07973219	0.04151974	0.03454995	0.168328	0.00789706	0.03831618	0.28105731	0.22242999	0.10392853	0.01034784	0.04617503	0.04731096
**Cameroon_g**	0.29018009	0.13328601	0.04420849	0.0020872	0.03780248		0.04084238	-2.37E-05	0.01058246	0.0645813	0.07391735	0.10686795	0.05292106	0.01495788	0.11146111	0.0019968	0.01585088	0.13933299	0.04468449	-0.0001602	0.25806717	0.19451472	0.06862239	0.04424555	0.02224971	0.00834668
**Central_col**	0.30700486	0.10426853	0.02238547	0.04467515	0.00922667	0.04084238		0.04333943	0.05211864	0.04065332	0.11117404	0.14641291	0.02228363	0.05726353	0.09220607	0.04436083	0.04166669	0.17194292	0.02349838	0.04212422	0.28437829	0.22585963	0.1063786	0.02455407	0.05320095	0.05043055
**Central_gam**	0.2920514	0.13628901	0.04627125	0.00190123	0.03993437	-2.37E-05	0.04333943		0.01170854	0.06681656	0.07421896	0.11118045	0.05513064	0.01554347	0.11355941	0.00279205	0.01656548	0.14167969	0.04771959	0.0005132	0.25989415	0.19710959	0.06951499	0.04679294	0.02249053	0.00845133
**Congo_gam**	0.30395078	0.1433981	0.05616128	0.01301965	0.04915211	0.01058246	0.05211864	0.01170854		0.07534	0.08467769	0.12065711	0.06463178	0.02711898	0.12276229	0.01361031	0.02719654	0.15078312	0.05678782	0.01161318	0.27045268	0.20577409	0.07992482	0.05658191	0.03345422	0.01931894
**Cote_col**	0.32408728	0.15351862	0.01593056	0.066866	0.02659763	0.0645813	0.04065332	0.06681656	0.07534		0.13296042	0.16818687	0.01942791	0.07922309	0.06001572	0.06787641	0.05517486	0.19175868	0.01865449	0.06390715	0.30138279	0.24410526	0.12892292	0.02839299	0.06731194	0.07381118
**Equatorial_g**	0.3840922	0.20057307	0.11454943	0.07597208	0.10846292	0.07391735	0.11117404	0.07421896	0.08467769	0.13296042		0.176332	0.12193131	0.08966876	0.17850722	0.07540204	0.08772839	0.2054948	0.11616025	0.07500865	0.32551946	0.26078002	0.1370191	0.11575517	0.09518996	0.08084496
**Gabon_gam**	0.41923653	0.2359484	0.14963107	0.113655	0.14395158	0.10686795	0.14641291	0.11118045	0.12065711	0.16818687	0.176332		0.15711481	0.12576286	0.21233321	0.11379088	0.12586353	0.21081842	0.14978621	0.1119786	0.33904212	0.2683433	0.14695972	0.14971028	0.13157784	0.10941612
**Ghana_col**	0.31074863	0.14161122	0.01208154	0.05546627	0.01310692	0.05292106	0.02228363	0.05513064	0.06463178	0.01942791	0.12193131	0.15711481		0.06864951	0.07233326	0.0563007	0.04169869	0.18103317	0.01368845	0.05279	0.2930004	0.23440898	0.1173002	0.01541663	0.05458119	0.06225252
**Ghana_gam**	0.30904664	0.14960597	0.05971987	0.01303225	0.05409953	0.01495788	0.05726353	0.01554347	0.02711898	0.07922309	0.08966876	0.12576286	0.06864951		0.12607359	0.01526965	0.0305796	0.15840322	0.06069245	0.01437307	0.27446883	0.21242874	0.08661702	0.06023234	0.03729464	0.02595926
**Guinea_col**	0.3827524	0.20363615	0.0682156	0.11389228	0.07973219	0.11146111	0.09220607	0.11355941	0.12276229	0.06001572	0.17850722	0.21233321	0.07233326	0.12607359		0.11511988	0.10112605	0.23831186	0.06942145	0.11075944	0.34981395	0.29009678	0.17555538	0.07709453	0.11324704	0.12034696
**Guinea_gam**	0.29374503	0.1359417	0.04792991	-3.97E-05	0.04151974	0.0019968	0.04436083	0.00279205	0.01361031	0.06787641	0.07540204	0.11379088	0.0563007	0.01526965	0.11511988		0.01763342	0.14437855	0.04833264	-0.0002312	0.26206647	0.20008482	0.07316344	0.04847893	0.02427967	0.01150956
**Guinea-Biss**	0.29547895	0.14284468	0.03751076	0.01742766	0.03454995	0.01585088	0.04166669	0.01656548	0.02719654	0.05517486	0.08772839	0.12586353	0.04169869	0.0305796	0.10112605	0.01763342		0.15482474	0.03810241	0.01540823	0.270243	0.20805967	0.08546959	0.02872419	0.00758587	0.0245602
**Kenya_gam**	0.43175084	0.26883025	0.17297418	0.14494354	0.168328	0.13933299	0.17194292	0.14167969	0.15078312	0.19175868	0.2054948	0.21081842	0.18103317	0.15840322	0.23831186	0.14437855	0.15482474		0.17368459	0.14376931	0.23054466	0.10296542	0.02501652	0.17403379	0.16212014	0.10414901
**Mali_col**	0.30635301	0.1366132	0.001737	0.0483443	0.00789706	0.04468449	0.02349838	0.04771959	0.05678782	0.01865449	0.11616025	0.14978621	0.01368845	0.06069245	0.06942145	0.04833264	0.03810241	0.17368459		0.04454956	0.28714689	0.22790313	0.10954607	0.01200947	0.05000153	0.05418887
**Mali_gam**	0.29018396	0.13412442	0.04366511	-0.0003485	0.03831618	-0.0001602	0.04212422	0.0005132	0.01161318	0.06390715	0.07500865	0.1119786	0.05279	0.01437307	0.11075944	-0.0002312	0.01540823	0.14376931	0.04454956		0.26030929	0.19818828	0.07164225	0.04471818	0.02171065	0.00957996
**Mayotte_gan**	0.63378162	0.38354765	0.28636356	0.26246899	0.28105731	0.25806717	0.28437829	0.25989415	0.27045268	0.30138279	0.32551946	0.33904212	0.2930004	0.27446883	0.34981395	0.26206647	0.270243	0.23054466	0.28714689	0.26030929		0.29126896	0.19730909	0.28824258	0.27883636	0.23387507
**Mozambique**	0.52255369	0.32197863	0.22674105	0.19923801	0.22242999	0.19451472	0.22585963	0.19710959	0.20577409	0.24410526	0.26078002	0.2683433	0.23440898	0.21242874	0.29009678	0.20008482	0.20805967	0.10296542	0.22790313	0.19818828	0.29126896		0.09596355	0.22815266	0.2164502	0.16272859
**Tanzania_ga**	0.34599189	0.20070187	0.10862156	0.07362196	0.10392853	0.06862239	0.1063786	0.06951499	0.07992482	0.12892292	0.1370191	0.14695972	0.1173002	0.08661702	0.17555538	0.07316344	0.08546959	0.02501652	0.10954607	0.07164225	0.19730909	0.09596355		0.10927546	0.0927776	0.03845894
**TheGambia_**	0.30477741	0.1402187	0.01063285	0.04716338	0.01034784	0.04424555	0.02455407	0.04679294	0.05658191	0.02839299	0.11575517	0.14971028	0.01541663	0.06023234	0.07709453	0.04847893	0.02872419	0.17403379	0.01200947	0.04471818	0.28824258	0.22815266	0.10927546		0.04103741	0.05409712
**TheGambia_**	0.30599453	0.15177958	0.0494066	0.0235359	0.04617503	0.02224971	0.05320095	0.02249053	0.03345422	0.06731194	0.09518996	0.13157784	0.05458119	0.03729464	0.11324704	0.02427967	0.00758587	0.16212014	0.05000153	0.02171065	0.27883636	0.2164502	0.0927776	0.04103741		0.03078127
**Uganda_gam**	0.29181963	0.14290916	0.05328676	0.01141785	0.04731096	0.00834668	0.05043055	0.00845133	0.01931894	0.07381118	0.08084496	0.10941612	0.06225252	0.02595926	0.12034696	0.01150956	0.0245602	0.10414901	0.05418887	0.00957996	0.23387507	0.16272859	0.03845894	0.05409712	0.03078127	

Supplementary Table S3 – Ancestry Info

**Chromoso**	**Position**	**An. gambi**	**An. coluzzi**
2R	7881405	C	A
2R	7978367	G	A
2R	8280785	T	A
2R	8307978	G	A
2R	8331032	G	A
2R	8369065	G	A
2R	8390985	C	A
2R	8395489	T	G
2R	8400296	G	A
2R	8416418	C	A
2R	8500131	G	A
2R	9641935	T	C
2R	11060650	C	T
2R	11187397	A	T
2R	11231237	G	A
2R	11232433	G	A
2R	11232589	T	A
2R	11244622	G	C
2R	17867695	T	C
2R	23702549	T	C
2R	28745760	T	G
2R	29027132	A	G
2R	29174959	C	A
2R	29194643	G	C
2R	38387409	T	A
2R	38412280	C	T
2R	39103333	C	T
2R	39992634	C	T
2R	45706992	A	T
2R	46099288	T	A
2R	46519094	A	G
2R	46956203	G	A
2R	47705614	C	A
2R	47711810	T	C
2R	47880229	C	A
2R	48625637	T	C
2R	48813932	T	G
2R	48814437	G	A
2R	49322120	A	G
2R	49732367	T	A
2R	49930642	G	T
2R	50352269	A	T
2R	52110509	G	A

2R	54083807	T	G
2R	55283824	C	T
2R	58748204	T	G
2R	58793509	G	C
2R	58899136	C	A
2R	59071740	A	G
2R	59073852	C	T
2R	60987243	T	A
2R	61010151	T	C
2R	61403855	T	C
2R	61455401	T	A
2L	181564	G	A
2L	210286	T	G
2L	356421	T	C
2L	441325	C	A
2L	456831	T	G
2L	494285	G	T
2L	741782	T	C
2L	742146	T	G
2L	743371	T	C
2L	770749	C	A
2L	771465	T	A
2L	772056	T	C
2L	856860	T	C
2L	927247	C	A
2L	933927	T	A
2L	955705	T	A
2L	955907	T	A
2L	987059	T	C
2L	1003551	T	C
2L	1057910	G	A
2L	1059325	T	C
2L	1156611	C	G
2L	1205929	T	C
2L	1275323	G	C
2L	1275498	T	A
2L	1283177	T	G
2L	1299662	T	C
2L	1328734	G	A
2L	1329290	T	G
2L	1330010	G	C
2L	1330524	G	A
2L	1418210	T	C
2L	1520251	T	A
2L	1534525	G	A
2L	1535842	C	A
2L	1543727	T	A

2L	1549512	C	A
2L	1566103	T	G
2L	1566702	T	A
2L	1569012	G	C
2L	1570427	G	C
2L	1571929	T	C
2L	1572122	G	A
2L	1574428	T	G
2L	1629562	T	G
2L	1773285	G	A
2L	1774816	T	G
2L	1776348	G	C
2L	1823373	T	C
2L	1834975	T	C
2L	1929057	C	A
2L	1929179	G	A
2L	1936042	G	A
2L	1947574	G	A
2L	2005557	T	G
2L	2049973	G	A
2L	2050540	T	G
2L	2050720	C	A
2L	2053999	T	C
2L	2076066	C	A
2L	2076374	T	G
2L	2079153	T	A
2L	2080181	C	A
2L	2081855	T	A
2L	2081947	C	A
2L	2108038	T	C
2L	2120222	T	C
2L	2121185	C	A
2L	2121209	T	G
2L	2122005	T	C
2L	2250268	T	C
2L	2288180	G	A
2L	2288436	C	A
2L	2381386	G	A
2L	2400620	T	A
2L	2423222	T	C
2L	2433476	T	C
2L	2433987	G	A
2L	2435532	G	A
2L	2439735	T	G
2L	2440600	G	A
2L	2681107	G	A
2L	2693637	T	A

2L	2729028	C	A
2L	4103472	T	A
2L	9239350	G	A
2L	10976291	T	C
2L	11245789	G	A
2L	11272048	T	G
2L	11475283	T	A
2L	11533813	T	C
2L	11554511	C	A
3R	27885	T	G
3R	87604	T	A
3R	105070	G	A
3R	108793	T	C
3R	455087	C	A
3R	455763	T	G
3R	459068	T	C
3R	486381	G	A
3R	28305711	G	A
3R	39374494	T	G
3R	41100730	T	G
3R	41394277	T	C
3R	41394921	T	G
3R	41463100	T	A
3R	41643924	G	A
3R	41704803	T	G
3R	42696964	C	A
3R	43382452	G	A
3R	46241353	C	A
3R	48921265	A	C
3R	48931724	C	A
3R	50142176	G	A
3R	50286348	T	C
3R	51005327	T	G
3R	52048223	T	A
3R	52048537	A	T
3R	52049336	T	C
3R	52049573	T	C
3R	52106373	G	C
3R	52112632	A	C
3R	52207724	G	T
3R	52212302	T	C
3R	52219184	T	C
3R	52225249	G	A
3R	52225294	T	G
3R	52247196	A	C
3R	52349404	G	A
3R	52356711	T	G

3R	52361157	G	A
3R	52422375	T	A
3R	52426862	A	G
3R	52486781	C	T
3R	52531329	C	A
3R	52614727	T	C
3R	52995713	T	G
3R	53082869	A	T
3R	53091720	C	A
3R	53093327	T	A
3L	67314	C	G
3L	77072	G	A
3L	82544	T	A
3L	122238	A	G
3L	122297	C	T
3L	122399	G	T
3L	122526	T	G
3L	122834	C	T
3L	182770	C	A
3L	302443	A	G
3L	304422	T	A
3L	304874	G	A
3L	331759	T	G
3L	344250	T	C
3L	344820	T	A
3L	1595454	A	G
3L	8593285	A	C
3L	8594715	C	G
3L	9515524	C	T
3L	9663677	A	T
3L	9667929	A	G
3L	9697405	G	T
3L	10882689	G	A
3L	10913379	T	G
X	4144	A	C
X	43831	C	T
X	15100037	T	C
X	15102030	T	G
X	15102258	T	A
X	15122298	C	A
X	15122738	G	C
X	15123143	T	C
X	15124440	G	A
X	15152322	T	G
X	15263921	T	A
X	15265556	G	A
X	15469805	T	C

X	15636260	C	A
X	15654817	G	C
X	15902143	T	A
X	15904818	G	C
X	15931132	G	A
X	15932344	G	A
X	15933212	T	C
X	15935327	T	C
X	15937755	T	A
X	16025283	C	A
X	16077738	G	A
X	17107813	T	A
X	17109956	T	A
X	17200215	G	A
X	17201100	T	A
X	17203086	T	C
X	17211289	G	A
X	17371331	G	A
X	17413658	G	A
X	17647028	C	A
X	17653571	G	A
X	17655806	T	G
X	17673204	T	C
X	17678530	G	A
X	17691764	T	C
X	17707969	T	C
X	17709956	G	A
X	17750600	G	A
X	17756330	G	A
X	17757534	A	G
X	17758240	T	C
X	17767738	T	C
X	17779517	T	C
X	17782492	T	A
X	17802089	T	C
X	17804078	T	C
X	17818460	G	C
X	17819878	T	C
X	17820570	C	A
X	17821599	T	A
X	17822430	T	A
X	17823362	C	A
X	17829586	G	A
X	17839055	C	A
X	17843560	G	A
X	17854530	G	A
X	17854770	T	C

X	17855890	T	G
X	17863952	T	G
X	17870231	G	A
X	17877673	G	A
X	17883470	G	A
X	17892553	T	C
X	17906226	T	A
X	17907713	T	A
X	17919967	T	C
X	17921848	G	A
X	17922361	T	A
X	17922652	C	A
X	17925859	C	A
X	17930773	T	A
X	17930970	G	C
X	17935016	G	A
X	17936437	G	A
X	17950636	T	C
X	17954268	T	C
X	17968899	T	A
X	17974469	G	A
X	17974772	T	A
X	18004091	T	C
X	18013323	T	C
X	18015016	T	A
X	18031772	G	A
X	18037533	T	C
X	18043476	C	A
X	18046469	G	A
X	18055878	G	A
X	18057451	T	C
X	18064888	C	A
X	18069465	T	A
X	18080014	T	C
X	18081652	T	C
X	18093475	C	A
X	18098910	C	A
X	18102566	T	C
X	18144827	C	A
X	18145980	T	A
X	18146820	T	C
X	18151779	G	A
X	18164515	C	A
X	18175646	G	C
X	18179121	T	G
X	18179484	T	C
X	18193109	T	G

X	18213254	C	A
X	18231178	G	A
X	18233449	T	A
X	18243004	T	A
X	18244075	G	A
X	18244214	G	A
X	18255024	T	C
X	18260913	T	A
X	18296187	G	A
X	18303117	G	C
X	18320057	T	C
X	18351413	G	A
X	18358985	T	C
X	18359371	T	A
X	18372750	T	G
X	18372852	G	C
X	18372890	T	G
X	18376928	T	C
X	18405057	C	A
X	18405644	T	G
X	18415090	T	G
X	18416362	T	C
X	18418071	G	A
X	18419070	T	G
X	18439071	T	C
X	18451087	T	C
X	18451490	T	A
X	18462956	T	C
X	18465552	T	C
X	18474471	T	A
X	18483223	T	C
X	18483308	G	C
X	18488749	T	G
X	18494213	C	A
X	18503755	T	C
X	18506770	T	A
X	18514224	T	A
X	18534685	G	A
X	18535307	T	C
X	18541695	T	C
X	18545950	T	C
X	18552400	C	A
X	18553766	T	C
X	18561007	G	A
X	18588345	T	A
X	18597532	T	G
X	18616815	T	C

X	18619378	G	A
X	18626514	C	A
X	18633609	T	A
X	18663447	G	A
X	18664320	G	A
X	18666391	T	C
X	18666995	G	A
X	18667110	T	C
X	18673482	G	C
X	18681536	T	G
X	18709711	G	A
X	18715423	T	C
X	18721037	T	C
X	18733692	T	A
X	18736537	G	A
X	18738010	T	C
X	18738227	T	C
X	18739073	C	A
X	18744530	T	G
X	18751262	T	C
X	18751643	G	A
X	18752247	T	C
X	18756071	G	A
X	18756626	C	A
X	18756937	G	C
X	18758300	T	C
X	18764064	T	A
X	18768526	C	A
X	18769165	T	A
X	18772627	T	A
X	18776386	T	A
X	18777403	G	C
X	18778473	T	C
X	18778618	G	C
X	18779655	G	C
X	18782469	T	A
X	18803767	T	C
X	18828121	T	A
X	18841196	T	A
X	18850598	G	A
X	18850788	T	C
X	18855100	C	A
X	18875872	T	G
X	18876416	T	C
X	18883085	T	A
X	18883338	T	C
X	18888979	T	A

X	18892658	G	A
X	18895846	T	C
X	18985260	G	C
X	18986203	T	A
X	19005798	G	A
X	19117756	G	A
X	19154757	T	C
X	19174276	T	G
X	19174735	G	C
X	19208510	T	C
X	19227341	T	C
X	19263223	C	A
X	19263806	T	C
X	19275911	T	A
X	19300779	T	C
X	19301398	G	A
X	19305628	T	C
X	19440618	T	A
X	19519687	G	A
X	19529503	T	C
X	19529806	T	C
X	19554201	C	A
X	19555085	G	A
X	19569364	T	G
X	19569839	G	C
X	19605776	T	C
X	19609315	T	A
X	19612071	T	C
X	19619051	T	C
X	19627490	T	C
X	19630571	G	A
X	19631429	T	C
X	19631625	G	C
X	19632267	G	A
X	19633607	T	C
X	19633780	T	G
X	19640247	G	A
X	19641255	G	A
X	19643251	G	A
X	19648745	G	A
X	19649825	T	G
X	19650465	C	A
X	19653831	G	A
X	19655218	C	A
X	19658441	G	A
X	19659462	C	A
X	19661079	G	A

X	19668644	G	A
X	19671378	T	A
X	19671719	G	A
X	19704860	T	C
X	19705887	G	C
X	19706296	T	G
X	19710357	T	C
X	19712176	T	G
X	19712866	C	A
X	19716539	T	C
X	19725156	G	A
X	19731241	T	A
X	19732984	T	A
X	19735812	T	A
X	19737928	G	A
X	19770048	T	C
X	19771211	G	A
X	19771979	G	A
X	19773913	T	C
X	19776018	T	C
X	19792887	G	A
X	19804741	G	A
X	19808574	T	G
X	19817906	T	G
X	19818055	T	A
X	19818982	T	A
X	19819412	T	A
X	19824714	G	A
X	19825227	G	C
X	19825246	T	C
X	19835133	T	A
X	19836117	G	A
X	19837576	G	C
X	19848422	G	A
X	19849243	C	A
X	19849804	T	A
X	19850268	T	A
X	19853409	T	C
X	19853649	T	C
X	19854380	T	C
X	19855512	G	C
X	19857473	T	C
X	19865560	T	C
X	19865723	C	A
X	19866581	G	A
X	19869654	G	A
X	19873216	G	A

X	19878888	T	A
X	19889029	T	A
X	19889093	T	C
X	19907496	G	C
X	19907655	T	C
X	19926288	G	A
X	19926762	G	A
X	19936493	G	A
X	19963776	T	G
X	19970630	A	G
X	19971453	T	G
X	19972120	T	A
X	19984239	T	G
X	19989651	G	A
X	19989942	G	A
X	19991095	G	A
X	19996358	T	A
X	19996699	G	A
X	20007133	T	C
X	20009203	G	A
X	20010584	C	A
X	20011785	T	C
X	20011891	T	C
X	20014696	C	A
X	20015293	G	C
X	20015634	T	G
X	20016494	T	C
X	20020741	T	A
X	20063553	G	A
X	20103469	C	A
X	20117226	T	C
X	20122753	G	A
X	20122967	G	A
X	20128328	T	C
X	20128465	T	A
X	20128719	T	G
X	20129288	T	G
X	20136659	T	C
X	20138331	G	A
X	20143470	T	A
X	20147987	T	C
X	20151732	G	A
X	20154269	T	A
X	20155233	G	T
X	20228797	C	A
X	20314336	G	A
X	20360797	T	C

X	20364269	T	G
X	20390449	C	A
X	20390601	T	G
X	20408988	T	C
X	20509533	G	C
X	20589835	T	A
X	20636715	T	C
X	20640231	G	A
X	20697661	T	C
X	20708775	T	C
X	20749100	C	A
X	20759443	T	C
X	20955606	G	A
X	20970988	T	C
X	20981685	G	A
X	20996337	T	A
X	21002147	T	C
X	21070424	C	A
X	21157981	C	A
X	21158253	T	A
X	21160323	T	G
X	21160773	T	A
X	21184030	T	C
X	21312932	T	A
X	21338102	T	C
X	21338291	G	A
X	21349307	T	G
X	21356202	T	A
X	21360274	T	A
X	21366870	T	G
X	21449903	T	G
X	21450550	G	A
X	21450774	T	A
X	21570512	T	G
X	21570867	T	A
X	21571300	T	C
X	21571781	T	A
X	21575124	C	A
X	21626163	T	C
X	21626338	T	C
X	21673523	G	A
X	21717027	T	A
X	21718625	T	A
X	21721801	G	C
X	21829550	G	A
X	21833728	T	C
X	22103050	C	A

X	22104143	T	A
X	22104513	T	C
X	22111379	G	A
X	22111592	T	C
X	22113822	T	C
X	22118497	T	C
X	22159812	G	C
X	22163586	T	C
X	22164043	T	C
X	22164752	T	A
X	22165570	G	A
X	22168355	C	A
X	22185114	T	C
X	22198101	T	A
X	22200639	G	A
X	22208526	G	A
X	22231151	G	A
X	22234030	G	A
X	22236887	T	A
X	22292977	G	C
X	22312208	C	A
X	22315938	T	A
X	22316162	G	A
X	22366774	G	A
X	22390324	T	G
X	22390361	C	A
X	22390982	T	C
X	22410608	G	A
X	22425428	T	A
X	22475989	G	A
X	22496458	T	C
X	22497394	G	A
X	22509931	T	G
X	22540496	T	A
X	22540777	G	C
X	22541551	G	A
X	22542908	T	C
X	22625335	C	A
X	22748466	G	A
X	22777161	T	C
X	22784625	C	A
X	22798402	T	A
X	22798449	G	A
X	22798648	C	A
X	22798890	G	A
X	22812582	T	C
X	22818209	T	A

X	22850158	G	A
X	22851469	T	C
X	22938882	G	A
X	22939198	G	A
X	22944596	G	A
X	23099740	G	C
X	23099906	T	C
X	23220106	G	A
X	23220968	G	C
X	23222641	G	C
X	23277649	C	A
X	23291595	T	C
X	23335699	G	C
X	23455776	C	A
X	23467702	G	A
X	23468221	G	C
X	23468268	T	A
X	23469565	T	C
X	23477824	T	C
X	23569262	T	G
X	23589842	T	A
X	23590469	G	A
X	23602240	T	G
X	23799054	T	A
X	23799458	G	A
X	23801326	T	A
X	23836150	T	A
X	23852135	T	A
X	23960686	C	A
X	23969255	T	C
X	23996455	T	A
X	24001181	C	A
X	24009756	C	A
X	24086167	G	C
X	24086825	T	C
X	24140221	T	A
X	24229846	G	A
X	24230141	T	G
X	24230996	T	A
X	24231230	T	A
X	24241386	T	A
X	24244054	T	C
X	24244165	G	A
X	24255864	G	A
X	24266355	T	G
X	24266728	T	A

Supplementary Table S4 – Metadata of subset of samples from Ag1000G used in this study, and sample ID and accession numbers of G3 strains.

Sample ID	Country	Location	Year of Collection	Latitude	Longitude	Species ID (AIMs)
AR0047-C	Angola	Luanda	2009	-8.884	13.302	coluzzii
AR0049-C	Angola	Luanda	2009	-8.884	13.302	coluzzii
AR0051-C	Angola	Luanda	2009	-8.884	13.302	coluzzii
AR0061-C	Angola	Luanda	2009	-8.884	13.302	coluzzii
AR0078-C	Angola	Luanda	2009	-8.884	13.302	coluzzii
AR0080-C	Angola	Luanda	2009	-8.884	13.302	coluzzii
AR0084-C	Angola	Luanda	2009	-8.884	13.302	coluzzii
AR0097-C	Angola	Luanda	2009	-8.884	13.302	coluzzii
AR0072-C	Angola	Luanda	2009	-8.884	13.302	coluzzii
AR0095-C	Angola	Luanda	2009	-8.884	13.302	coluzzii
AB0096-C	Burkina Faso	Bana Village	2012	11.233	-4.472	gambiae
AB0373-C	Burkina Faso	Bana Village	2014	11.233	-4.472	gambiae
AB0374-C	Burkina Faso	Bana Village	2014	11.233	-4.472	gambiae
AB0298-C	Burkina Faso	Monomtenga	2004	12.06	-1.17	gambiae
AB0299-C	Burkina Faso	Monomtenga	2004	12.06	-1.17	gambiae
AB0085-Cx	Burkina Faso	Pala	2012	11.151	-4.235	gambiae
AB0086-Cx	Burkina Faso	Pala	2012	11.151	-4.235	gambiae
AB0458-C	Burkina Faso	Pala	2014	11.151	-4.235	gambiae
AB0133-C	Burkina Faso	Souroukoudinga	2012	11.238	-4.235	gambiae
AB0504-C	Burkina Faso	Souroukoudinga	2014	11.238	-4.235	gambiae
AB0088-C	Burkina Faso	Bana Village	2012	11.233	-4.472	coluzzii
AB0089-Cx	Burkina Faso	Bana Village	2012	11.233	-4.472	coluzzii
AB0326-C	Burkina Faso	Bana Village	2014	11.233	-4.472	coluzzii
AB0327-C	Burkina Faso	Bana Village	2014	11.233	-4.472	coluzzii
AB0204-C	Burkina Faso	Pala	2012	11.151	-4.235	coluzzii
AB0257-C	Burkina Faso	Pala	2012	11.151	-4.235	coluzzii
AB0132-C	Burkina Faso	Souroukoudinga	2012	11.238	-4.235	coluzzii
AB0137-Cx	Burkina Faso	Souroukoudinga	2012	11.238	-4.235	coluzzii
AB0503-C	Burkina Faso	Souroukoudinga	2014	11.238	-4.235	coluzzii
AB0505-C	Burkina Faso	Souroukoudinga	2014	11.238	-4.235	coluzzii
BP0010-C	Democratic Republic of	Gbadolite	2015	4.283	21.017	gambiae
BP0012-C	Democratic Republic of	Gbadolite	2015	4.283	21.017	gambiae
BP0015-C	Democratic Republic of	Gbadolite	2015	4.283	21.017	gambiae
BP0018-C	Democratic Republic of	Gbadolite	2015	4.283	21.017	gambiae
BP0019-C	Democratic Republic of	Gbadolite	2015	4.283	21.017	gambiae
BP0020-C	Democratic Republic of	Gbadolite	2015	4.283	21.017	gambiae
BP0022-C	Democratic Republic of	Gbadolite	2015	4.283	21.017	gambiae
BP0024-C	Democratic Republic of	Gbadolite	2015	4.283	21.017	gambiae
BP0025-C	Democratic Republic of	Gbadolite	2015	4.283	21.017	gambiae
BP0026-C	Democratic Republic of	Gbadolite	2015	4.283	21.017	gambiae
AY0072-C	Cote d'Ivoire	Tiassale	2012	5.898	-4.823	coluzzii
AY0062-C	Cote d'Ivoire	Tiassale	2012	5.898	-4.823	coluzzii
AY0055-C	Cote d'Ivoire	Tiassale	2012	5.898	-4.823	coluzzii
AY0034-C	Cote d'Ivoire	Tiassale	2012	5.898	-4.823	coluzzii
AY0078-C	Cote d'Ivoire	Tiassale	2012	5.898	-4.823	coluzzii
AY0033-C	Cote d'Ivoire	Tiassale	2012	5.898	-4.823	coluzzii
AY0064-C	Cote d'Ivoire	Tiassale	2012	5.898	-4.823	coluzzii
AY0032-C	Cote d'Ivoire	Tiassale	2012	5.898	-4.823	coluzzii
AY0067-C	Cote d'Ivoire	Tiassale	2012	5.898	-4.823	coluzzii
AY0059-C	Cote d'Ivoire	Tiassale	2012	5.898	-4.823	coluzzii
BK0001-C	Central African Republi	Bangui	1993	4.367	18.583	gambiae
BK0005-C	Central African Republi	Bangui	1993	4.367	18.583	gambiae
BK0009-C	Central African Republi	Bangui	1994	4.367	18.583	gambiae
BK0010-C	Central African Republi	Bangui	1994	4.367	18.583	gambiae
BK0011-C	Central African Republi	Bangui	1994	4.367	18.583	gambiae
BK0013-C	Central African Republi	Bangui	1994	4.367	18.583	gambiae
BK0016-C	Central African Republi	Bangui	1994	4.367	18.583	gambiae
BK0018-C	Central African Republi	Bangui	1994	4.367	18.583	gambiae
BK0019-C	Central African Republi	Bangui	1994	4.367	18.583	gambiae
BK0022-C	Central African Republi	Bangui	1994	4.367	18.583	gambiae
BK0002-C	Central African Republi	Bangui	1993	4.367	18.583	coluzzii
BK0003-C	Central African Republi	Bangui	1993	4.367	18.583	coluzzii
BK0006-C	Central African Republi	Bangui	1993	4.367	18.583	coluzzii

BK0007-C	Central African Republi	Bangui	1993	4.367	18.583	coluzzii
BK0008-C	Central African Republi	Bangui	1993	4.367	18.583	coluzzii
BK0014-C	Central African Republi	Bangui	1994	4.367	18.583	coluzzii
BK0015-C	Central African Republi	Bangui	1994	4.367	18.583	coluzzii
BK0017-C	Central African Republi	Bangui	1994	4.367	18.583	coluzzii
BK0021-C	Central African Republi	Bangui	1994	4.367	18.583	coluzzii
BK0024-C	Central African Republi	Bangui	1994	4.367	18.583	coluzzii
AQ0009-C	Equatorial Guinea	Bioko	2002	3.7	8.7	gambiae
AQ0001-C	Equatorial Guinea	Bioko	2002	3.7	8.7	gambiae
AQ0002-C	Equatorial Guinea	Bioko	2002	3.7	8.7	gambiae
AQ0005-C	Equatorial Guinea	Bioko	2002	3.7	8.7	gambiae
AQ0004-C	Equatorial Guinea	Bioko	2002	3.7	8.7	gambiae
AQ0015-C	Equatorial Guinea	Bioko	2002	3.7	8.7	gambiae
AQ0011-C	Equatorial Guinea	Bioko	2002	3.7	8.7	gambiae
AQ0012-C	Equatorial Guinea	Bioko	2002	3.7	8.7	gambiae
AQ0014-C	Equatorial Guinea	Bioko	2002	3.7	8.7	gambiae
AQ0013-C	Equatorial Guinea	Bioko	2002	3.7	8.7	gambiae
AS0001-C	Gabon	Libreville	2000	0.384	9.455	gambiae
AS0002-Cx	Gabon	Libreville	2000	0.384	9.455	gambiae
AS0003-C	Gabon	Libreville	2000	0.384	9.455	gambiae
AS0004-C	Gabon	Libreville	2000	0.384	9.455	gambiae
AS0005-C	Gabon	Libreville	2000	0.384	9.455	gambiae
AS0006-C	Gabon	Libreville	2000	0.384	9.455	gambiae
AS0007-C	Gabon	Libreville	2000	0.384	9.455	gambiae
AS0008-Cx	Gabon	Libreville	2000	0.384	9.455	gambiae
AS0009-C	Gabon	Libreville	2000	0.384	9.455	gambiae
AS0010-C	Gabon	Libreville	2000	0.384	9.455	gambiae
AG0423-CW	Gambia, The	Kalataba	2012	13.55	-15.617	coluzzii
AG0200-C	Gambia, The	Njabakunda	2011	13.55	-15.9	coluzzii
AG0209-C	Gambia, The	Njabakunda	2011	13.55	-15.9	coluzzii
AG0443-CW	Gambia, The	Sare Samba Sowe	2012	13.583	-15.9	coluzzii
AG0451-CW	Gambia, The	Sare Samba Sowe	2012	13.583	-15.9	coluzzii
AG0386-CW	Gambia, The	Tankular	2012	13.417	-16.033	coluzzii
AG0390-CW	Gambia, The	Tankular	2012	13.417	-16.033	coluzzii
AG0001-C	Gambia, The	Wali Kunda	2012	13.567	-14.917	coluzzii
AG0003-C	Gambia, The	Wali Kunda	2012	13.567	-14.917	coluzzii
AG0004-C	Gambia, The	Wali Kunda	2012	13.567	-14.917	coluzzii
AG0159-C	Gambia, The	Njabakunda	2011	13.55	-15.9	gambiae
AG0232-C	Gambia, The	Njabakunda	2011	13.55	-15.9	gambiae
AG0129-C	Gambia, The	Njabakunda	2011	13.55	-15.9	gambiae
AG0153-C	Gambia, The	Njabakunda	2011	13.55	-15.9	gambiae
AG0445-CW	Gambia, The	Sare Samba Sowe	2012	13.583	-15.9	gambiae
AG0446-CW	Gambia, The	Sare Samba Sowe	2012	13.583	-15.9	gambiae
AG0448-CW	Gambia, The	Sare Samba Sowe	2012	13.583	-15.9	gambiae
AG0450-CW	Gambia, The	Sare Samba Sowe	2012	13.583	-15.9	gambiae
AG0058-C	Gambia, The	Wali Kunda	2012	13.567	-14.917	gambiae
AG0074-C	Gambia, The	Wali Kunda	2012	13.567	-14.917	gambiae
AA0107-C	Ghana	Koforidua	2012	6.094	-0.261	coluzzii
AA0134-C	Ghana	Madina_West	2012	5.668	-0.219	coluzzii
AA0098-C	Ghana	Madina_West	2012	5.668	-0.219	coluzzii
AA0097-C	Ghana	Madina_West	2012	5.668	-0.219	coluzzii
AA0103-C	Ghana	Takoradi	2012	4.912	-1.774	coluzzii
AA0115-C	Ghana	Takoradi	2012	4.912	-1.774	coluzzii
AA0055-C	Ghana	Takoradi	2012	4.912	-1.774	coluzzii
AA0052-C	Ghana	Twifo Praso	2012	5.609	-1.549	coluzzii
AA0063-C	Ghana	Twifo Praso	2012	5.609	-1.549	coluzzii
AA0040-C	Ghana	Twifo Praso	2012	5.609	-1.549	coluzzii
AA0059-C	Ghana	Koforidua	2012	6.094	-0.261	gambiae
AA0106-C	Ghana	Koforidua	2012	6.094	-0.261	gambiae
AA0119-C	Ghana	Koforidua	2012	6.094	-0.261	gambiae
AA0105-C	Ghana	Koforidua	2012	6.094	-0.261	gambiae
AA0070-C	Ghana	Koforidua	2012	6.094	-0.261	gambiae
AA0050-C	Ghana	Madina_West	2012	5.668	-0.219	gambiae
AA0084-C	Ghana	Madina_West	2012	5.668	-0.219	gambiae
AA0060-C	Ghana	Madina_West	2012	5.668	-0.219	gambiae

AA0061-C	Ghana	Madina_West	2012	5.668	-0.219	gambiae
AA0133-C	Ghana	Madina_West	2012	5.668	-0.219	gambiae
AV0038-C	Guinea	Koundara	2012	8.48	-9.53	coluzzii
AV0040-C	Guinea	Koundara	2012	8.48	-9.53	coluzzii
AV0042-Cx	Guinea	Koundara	2012	8.48	-9.53	coluzzii
AV0046-Cx	Guinea	Koundara	2012	8.48	-9.53	coluzzii
AV0209-CW	Guinea	Koundara	2012	8.48	-9.53	coluzzii
AV0207-C	Guinea	Koundara	2012	8.48	-9.53	coluzzii
AV0220-C	Guinea	Koundara	2012	8.48	-9.53	coluzzii
AV0232-C	Guinea	Koundara	2012	8.48	-9.53	coluzzii
AV0149-C	Guinea	Koundara	2012	8.48	-9.53	coluzzii
AV0161-C	Guinea	Koundara	2012	8.48	-9.53	coluzzii
AV0025-C	Guinea	Koraboh	2012	9.28	-10.03	gambiae
AV0006-C	Guinea	Koraboh	2012	9.28	-10.03	gambiae
AV0008-C	Guinea	Koraboh	2012	9.28	-10.03	gambiae
AV0015-C	Guinea	Koraboh	2012	9.28	-10.03	gambiae
AV0010-C	Guinea	Koraboh	2012	9.28	-10.03	gambiae
AV0043-C	Guinea	Koundara	2012	8.48	-9.53	gambiae
AV0036-C	Guinea	Koundara	2012	8.48	-9.53	gambiae
AV0037-C	Guinea	Koundara	2012	8.48	-9.53	gambiae
AV0026-C	Guinea	Koundara	2012	8.48	-9.53	gambiae
AV0032-C	Guinea	Koundara	2012	8.48	-9.53	gambiae
AJ0023-C	Guinea-Bissau	Antula	2010	11.891	-15.582	gambiae
AJ0039-C	Guinea-Bissau	Antula	2010	11.891	-15.582	gambiae
AJ0056-C	Guinea-Bissau	Antula	2010	11.891	-15.582	gambiae
AJ0059-C	Guinea-Bissau	Antula	2010	11.891	-15.582	gambiae
AJ0120-C	Guinea-Bissau	Leibala	2010	12.272	-14.222	gambiae
AJ0121-C	Guinea-Bissau	Leibala	2010	12.272	-14.222	gambiae
AJ0122-C	Guinea-Bissau	Leibala	2010	12.272	-14.222	gambiae
AJ0131-C	Guinea-Bissau	Safim	2010	11.957	-15.649	gambiae
AJ0133-C	Guinea-Bissau	Safim	2010	11.957	-15.649	gambiae
AJ0138-C	Guinea-Bissau	Safim	2010	11.957	-15.649	gambiae
AK0177-C	Kenya	Kilifi	2000	-3.511	39.909	gambiae
AK0213-C	Kenya	Kilifi	2000	-3.511	39.909	gambiae
AK0235-C	Kenya	Kilifi	2000	-3.511	39.909	gambiae
AK0237-C	Kenya	Kilifi	2000	-3.511	39.909	gambiae
AK0238-C	Kenya	Kilifi	2000	-3.511	39.909	gambiae
AK0239-C	Kenya	Kilifi	2000	-3.511	39.909	gambiae
AK0274-C	Kenya	Kilifi	2000	-3.511	39.909	gambiae
AK0280-C	Kenya	Kilifi	2000	-3.511	39.909	gambiae
AK0062-C	Kenya	Kilifi	2012	-3.511	39.909	gambiae
AK0063-C	Kenya	Kilifi	2012	-3.511	39.909	gambiae
AZ0253-C	Mali	Bancoumana	2004	12.2	-8.2	gambiae
AZ0254-C	Mali	Bancoumana	2004	12.2	-8.2	gambiae
AZ0267-C	Mali	Fanzana	2004	13.2	-6.13	gambiae
AZ0263-C	Mali	Kela	2004	11.88	-8.45	gambiae
AV0263-C	Mali	Takan	2012	11.47	-8.33	gambiae
AV0330-C	Mali	Toumani Oulena	2012	10.83	-7.81	gambiae
AV0342-C	Mali	Toumani Oulena	2012	10.83	-7.81	gambiae
AZ0300-C	Mali	Kababougou	2014	12.89	-8.15	gambiae
AZ0336-C	Mali	Ouassorola	2014	12.9	-8.16	gambiae
AZ0484-CW	Mali	Tieneguebougou	2014	12.81	-8.08	gambiae
AZ0280-C	Mali	Douna	2004	13.21	-5.9	coluzzii
AZ0268-C	Mali	Fanzana	2004	13.2	-6.13	coluzzii
AZ0272-C	Mali	Moriboboougou	2004	12.69	-7.87	coluzzii
VBS02051-44	Mali	N'Gabakoro	2004	12.68	-7.84	coluzzii
AV0236-C	Mali	Takan	2012	11.47	-8.33	coluzzii
AV0366-C	Mali	Toumani Oulena	2012	10.83	-7.81	coluzzii
AV0340-C	Mali	Toumani Oulena	2012	10.83	-7.81	coluzzii
AZ0292-C	Mali	Kababougou	2014	12.89	-8.15	coluzzii
AZ0332-C	Mali	Ouassorola	2014	12.9	-8.16	coluzzii
AZ0425-CW	Mali	Tieneguebougou	2014	12.81	-8.08	coluzzii
AP0005-C	Mayotte	Bouyouni	2011	-12.738	45.142	gambiae
AP0002-C	Mayotte	Combani	2011	-12.779	45.143	gambiae
AP0008-C	Mayotte	Combani	2011	-12.779	45.143	gambiae

AP0021-C	Mayotte	Karihani Lake	2011	-12.797	45.122	gambiae
AP0022-C	Mayotte	Karihani Lake	2011	-12.797	45.122	gambiae
AP0030-C	Mayotte	Mont Benara	2011	-12.857	45.155	gambiae
AP0006-C	Mayotte	Mtsanga Charifou	2011	-12.991	45.156	gambiae
AP0017-Cx	Mayotte	Mtsanga Charifou	2011	-12.991	45.156	gambiae
AP0007-C	Mayotte	Mtsamboro Forest Rese	2011	-12.703	45.081	gambiae
AP0035-C	Mayotte	Sada	2011	-12.852	45.104	gambiae
BQ0122-C	Mozambique	Furvela	2003	-23.716	35.299	gambiae
BQ0123-C	Mozambique	Furvela	2003	-23.716	35.299	gambiae
BQ0125-C	Mozambique	Furvela	2003	-23.716	35.299	gambiae
BQ0046-C	Mozambique	Furvela	2004	-23.716	35.299	gambiae
BQ0047-C	Mozambique	Furvela	2004	-23.716	35.299	gambiae
BQ0049-C	Mozambique	Furvela	2004	-23.716	35.299	gambiae
BQ0050-C	Mozambique	Furvela	2004	-23.716	35.299	gambiae
BQ0051-C	Mozambique	Furvela	2004	-23.716	35.299	gambiae
BQ0052-C	Mozambique	Furvela	2004	-23.716	35.299	gambiae
BQ0053-C	Mozambique	Furvela	2004	-23.716	35.299	gambiae
BL0346-C	Tanzania	Muheza	2013	-4.94	38.948	gambiae
BL0347-C	Tanzania	Muheza	2013	-4.94	38.948	gambiae
BL0348-C	Tanzania	Muheza	2013	-4.94	38.948	gambiae
BL0350-C	Tanzania	Muheza	2013	-4.94	38.948	gambiae
BL0351-C	Tanzania	Muheza	2013	-4.94	38.948	gambiae
BL0063-C	Tanzania	Muleba	2015	-1.962	31.621	gambiae
BL0065-C	Tanzania	Muleba	2015	-1.962	31.621	gambiae
BL0078-C	Tanzania	Muleba	2015	-1.962	31.621	gambiae
BL0079-C	Tanzania	Muleba	2015	-1.962	31.621	gambiae
BL0101-C	Tanzania	Muleba	2015	-1.962	31.621	gambiae
AN0486-C	Cameroon	Badankali	2005	8.66	13.529	coluzzii
AN0487-C	Cameroon	Carrefour Poli	2005	8.534	13.53	coluzzii
AN0493-C	Cameroon	Gakle	2005	10.522	14.265	coluzzii
AN0492-C	Cameroon	Gamba	2005	8.098	13.599	coluzzii
AN0488-C	Cameroon	Gouna	2005	8.525	13.564	coluzzii
AN0460-C	Cameroon	Palama	2005	10.462	14.228	coluzzii
AN0485-C	Cameroon	Wouro Andre	2005	8.888	13.503	coluzzii
AN0633-CW	Cameroon	Campo	2013	2.367	9.817	coluzzii
AN0590-CW	Cameroon	Douala	2013	4.055	9.721	coluzzii
AN0544-CW	Cameroon	Lagdo	2013	9.049	13.656	coluzzii
VBS02014-44	Cameroon	Carrefour Nari	2005	8.801	13.52	gambiae
VBS02042-44	Cameroon	Beka Goto	2005	6.751	13.111	gambiae
AN0099-C	Cameroon	Gado Badzere	2009	5.747	14.442	gambiae
VBS02045-44	Cameroon	Avebe	2005	3.369	11.519	gambiae
VBS02038-44	Cameroon	Lougga Tapadi	2005	7.107	13.209	gambiae
AN0457-C	Cameroon	Lougol	2005	10.495	14.255	gambiae
VBS02020-44	Cameroon	Balda Bouri	2005	8.513	13.603	gambiae
VBS02048-44	Cameroon	Domb ├╒	2005	2.949	9.927	gambiae
AB0293-C	Cameroon	Doulougou	2005	10.425	14.242	gambiae
AN0016-C	Cameroon	Mayos	2009	4.341	13.558	gambiae
AC0204-C	Uganda	Kihihi	2012	-0.751	29.701	gambiae
AC0207-C	Uganda	Kihihi	2012	-0.751	29.701	gambiae
AC0208-C	Uganda	Kihihi	2012	-0.751	29.701	gambiae
AC0209-C	Uganda	Kihihi	2012	-0.751	29.701	gambiae
AC0210-C	Uganda	Kihihi	2012	-0.751	29.701	gambiae
AC0109-C	Uganda	Nagongera	2012	0.77	34.026	gambiae
AC0110-Cx	Uganda	Nagongera	2012	0.77	34.026	gambiae
AC0111-Cx	Uganda	Nagongera	2012	0.77	34.026	gambiae
AC0112-C	Uganda	Nagongera	2012	0.77	34.026	gambiae
AC0113-Cx	Uganda	Nagongera	2012	0.77	34.026	gambiae
AB0131-Cx	Burkina Faso	Bana Village	2012	11.233	-4.472	intermediate
AG0010-C	Gambia, The	Wali Kunda	2012	13.567	-14.917	intermediate
AG0016-C	Gambia, The	Wali Kunda	2012	13.567	-14.917	intermediate
AG0018-C	Gambia, The	Wali Kunda	2012	13.567	-14.917	intermediate
AG0019-C	Gambia, The	Wali Kunda	2012	13.567	-14.917	intermediate
AG0033-C	Gambia, The	Wali Kunda	2012	13.567	-14.917	intermediate
AG0040-C	Gambia, The	Wali Kunda	2012	13.567	-14.917	intermediate
AG0049-C	Gambia, The	Wali Kunda	2012	13.567	-14.917	intermediate

AG0060-C	Gambia, The	Wali Kunda	2012	13.567	-14.917	intermediate
AG0063-C	Gambia, The	Wali Kunda	2012	13.567	-14.917	intermediate
AG0064-C	Gambia, The	Wali Kunda	2012	13.567	-14.917	intermediate
AG0065-C	Gambia, The	Wali Kunda	2012	13.567	-14.917	intermediate
AG0071-C	Gambia, The	Wali Kunda	2012	13.567	-14.917	intermediate
AG0096-C	Gambia, The	Njabakunda	2011	13.55	-15.9	intermediate
AG0100-C	Gambia, The	Njabakunda	2011	13.55	-15.9	intermediate
AG0118-C	Gambia, The	Njabakunda	2011	13.55	-15.9	intermediate
AG0120-C	Gambia, The	Njabakunda	2011	13.55	-15.9	intermediate
AG0127-C	Gambia, The	Njabakunda	2011	13.55	-15.9	intermediate
AG0147-C	Gambia, The	Njabakunda	2011	13.55	-15.9	intermediate
AG0172-C	Gambia, The	Njabakunda	2011	13.55	-15.9	intermediate
AG0181-C	Gambia, The	Njabakunda	2011	13.55	-15.9	intermediate
AG0195-C	Gambia, The	Njabakunda	2011	13.55	-15.9	intermediate
AG0204-C	Gambia, The	Njabakunda	2011	13.55	-15.9	intermediate
AG0206-C	Gambia, The	Njabakunda	2011	13.55	-15.9	intermediate
AG0214-C	Gambia, The	Njabakunda	2011	13.55	-15.9	intermediate
AG0226-C	Gambia, The	Njabakunda	2011	13.55	-15.9	intermediate
AG0227-C	Gambia, The	Njabakunda	2011	13.55	-15.9	intermediate
AG0230-C	Gambia, The	Njabakunda	2011	13.55	-15.9	intermediate
AG0300-C	Gambia, The	Wali Kunda	2012	13.567	-14.917	intermediate
AG0301-C	Gambia, The	Wali Kunda	2012	13.567	-14.917	intermediate
AG0305-C	Gambia, The	Wali Kunda	2012	13.567	-14.917	intermediate
AG0307-C	Gambia, The	Wali Kunda	2012	13.567	-14.917	intermediate
AG0320-C	Gambia, The	Wali Kunda	2012	13.567	-14.917	intermediate
AG0344-C	Gambia, The	Wali Kunda	2012	13.567	-14.917	intermediate
AG0346-C	Gambia, The	Wali Kunda	2012	13.567	-14.917	intermediate
AG0360-C	Gambia, The	Wali Kunda	2012	13.567	-14.917	intermediate
AG0366-C	Gambia, The	Wali Kunda	2012	13.567	-14.917	intermediate
AG0369-C	Gambia, The	Wali Kunda	2012	13.567	-14.917	intermediate
AG0376-C	Gambia, The	Wali Kunda	2012	13.567	-14.917	intermediate
AG0382-CW	Gambia, The	Tankular	2012	13.417	-16.033	intermediate
AG0394-CW	Gambia, The	Tankular	2012	13.417	-16.033	intermediate
AG0404-CW	Gambia, The	Tankular	2012	13.417	-16.033	intermediate
AG0405-CW	Gambia, The	Tankular	2012	13.417	-16.033	intermediate
AG0410-CW	Gambia, The	Tankular	2012	13.417	-16.033	intermediate
AG0420-CW	Gambia, The	Tankular	2012	13.417	-16.033	intermediate
AG0454-CW	Gambia, The	Sare Samba Sowe	2012	13.583	-15.9	intermediate
AJ0024-C	Guinea-Bissau	Antula	2010	11.891	-15.582	intermediate
AJ0028-C	Guinea-Bissau	Antula	2010	11.891	-15.582	intermediate
AJ0032-C	Guinea-Bissau	Antula	2010	11.891	-15.582	intermediate
AJ0035-C	Guinea-Bissau	Antula	2010	11.891	-15.582	intermediate
AJ0036-C	Guinea-Bissau	Antula	2010	11.891	-15.582	intermediate
AJ0037-C	Guinea-Bissau	Antula	2010	11.891	-15.582	intermediate
AJ0043-C	Guinea-Bissau	Antula	2010	11.891	-15.582	intermediate
AJ0044-C	Guinea-Bissau	Antula	2010	11.891	-15.582	intermediate
AJ0045-C	Guinea-Bissau	Antula	2010	11.891	-15.582	intermediate
AJ0047-C	Guinea-Bissau	Antula	2010	11.891	-15.582	intermediate
AJ0051-C	Guinea-Bissau	Antula	2010	11.891	-15.582	intermediate
AJ0052-C	Guinea-Bissau	Antula	2010	11.891	-15.582	intermediate
AJ0060-C	Guinea-Bissau	Antula	2010	11.891	-15.582	intermediate
AJ0063-C	Guinea-Bissau	Antula	2010	11.891	-15.582	intermediate
AJ0064-C	Guinea-Bissau	Antula	2010	11.891	-15.582	intermediate
AJ0066-C	Guinea-Bissau	Antula	2010	11.891	-15.582	intermediate
AJ0068-C	Guinea-Bissau	Antula	2010	11.891	-15.582	intermediate
AJ0070-Cx	Guinea-Bissau	Antula	2010	11.891	-15.582	intermediate
AJ0074-C	Guinea-Bissau	Antula	2010	11.891	-15.582	intermediate
AJ0075-C	Guinea-Bissau	Antula	2010	11.891	-15.582	intermediate
AJ0076-C	Guinea-Bissau	Antula	2010	11.891	-15.582	intermediate
AJ0077-C	Guinea-Bissau	Antula	2010	11.891	-15.582	intermediate
AJ0078-C	Guinea-Bissau	Antula	2010	11.891	-15.582	intermediate
AJ0079-C	Guinea-Bissau	Antula	2010	11.891	-15.582	intermediate
AJ0080-C	Guinea-Bissau	Antula	2010	11.891	-15.582	intermediate
AJ0081-C	Guinea-Bissau	Antula	2010	11.891	-15.582	intermediate
AJ0084-C	Guinea-Bissau	Antula	2010	11.891	-15.582	intermediate

AJ0086-C	Guinea-Bissau	Antula	2010	11.891	-15.582	intermediate
AJ0087-C	Guinea-Bissau	Antula	2010	11.891	-15.582	intermediate
AJ0088-C	Guinea-Bissau	Antula	2010	11.891	-15.582	intermediate
AJ0090-C	Guinea-Bissau	Antula	2010	11.891	-15.582	intermediate
AJ0092-C	Guinea-Bissau	Antula	2010	11.891	-15.582	intermediate
AJ0093-C	Guinea-Bissau	Antula	2010	11.891	-15.582	intermediate
AJ0095-C	Guinea-Bissau	Antula	2010	11.891	-15.582	intermediate
AJ0096-C	Guinea-Bissau	Antula	2010	11.891	-15.582	intermediate
AJ0098-C	Guinea-Bissau	Antula	2010	11.891	-15.582	intermediate
AJ0099-C	Guinea-Bissau	Antula	2010	11.891	-15.582	intermediate
AJ0100-C	Guinea-Bissau	Antula	2010	11.891	-15.582	intermediate
AJ0101-Cx	Guinea-Bissau	Antula	2010	11.891	-15.582	intermediate
AJ0102-C	Guinea-Bissau	Antula	2010	11.891	-15.582	intermediate
AJ0103-C	Guinea-Bissau	Antula	2010	11.891	-15.582	intermediate
AJ0105-C	Guinea-Bissau	Antula	2010	11.891	-15.582	intermediate
AJ0109-C	Guinea-Bissau	Antula	2010	11.891	-15.582	intermediate
AJ0115-C	Guinea-Bissau	Antula	2010	11.891	-15.582	intermediate
AJ0116-C	Guinea-Bissau	Antula	2010	11.891	-15.582	intermediate
AJ0119-C	Guinea-Bissau	Antula	2010	11.891	-15.582	intermediate
AJ0128-C	Guinea-Bissau	Safim	2010	11.957	-15.649	intermediate
AJ0129-C	Guinea-Bissau	Safim	2010	11.957	-15.649	intermediate
AJ0130-C	Guinea-Bissau	Safim	2010	11.957	-15.649	intermediate
AJ0132-C	Guinea-Bissau	Safim	2010	11.957	-15.649	intermediate
AJ0134-C	Guinea-Bissau	Safim	2010	11.957	-15.649	intermediate
AJ0135-C	Guinea-Bissau	Safim	2010	11.957	-15.649	intermediate
AJ0136-C	Guinea-Bissau	Safim	2010	11.957	-15.649	intermediate
AJ0137-C	Guinea-Bissau	Safim	2010	11.957	-15.649	intermediate
AJ0139-C	Guinea-Bissau	Safim	2010	11.957	-15.649	intermediate
AJ0141-C	Guinea-Bissau	Safim	2010	11.957	-15.649	intermediate
AJ0142-C	Guinea-Bissau	Safim	2010	11.957	-15.649	intermediate
AJ0143-C	Guinea-Bissau	Safim	2010	11.957	-15.649	intermediate
AJ0144-C	Guinea-Bissau	Safim	2010	11.957	-15.649	intermediate
AJ0146-C	Guinea-Bissau	Safim	2010	11.957	-15.649	intermediate
AJ0147-C	Guinea-Bissau	Safim	2010	11.957	-15.649	intermediate
AJ0148-C	Guinea-Bissau	Safim	2010	11.957	-15.649	intermediate
AJ0149-C	Guinea-Bissau	Safim	2010	11.957	-15.649	intermediate
AJ0150-C	Guinea-Bissau	Safim	2010	11.957	-15.649	intermediate
AJ0151-C	Guinea-Bissau	Safim	2010	11.957	-15.649	intermediate
AJ0152-C	Guinea-Bissau	Safim	2010	11.957	-15.649	intermediate
AJ0153-C	Guinea-Bissau	Safim	2010	11.957	-15.649	intermediate
AJ0154-C	Guinea-Bissau	Safim	2010	11.957	-15.649	intermediate
AJ0156-C	Guinea-Bissau	Safim	2010	11.957	-15.649	intermediate
AJ0157-C	Guinea-Bissau	Safim	2010	11.957	-15.649	intermediate
AJ0158-C	Guinea-Bissau	Safim	2010	11.957	-15.649	intermediate
AJ0159-C	Guinea-Bissau	Safim	2010	11.957	-15.649	intermediate
AJ0161-C	Guinea-Bissau	Safim	2010	11.957	-15.649	intermediate
AV0007-C	Guinea	Koraboh	2012	9.28	-10.03	intermediate
AV0126-CW	Guinea	Koraboh	2012	9.28	-10.03	intermediate
AV0331-C	Mali	Toumani Oulena	2012	10.83	-7.81	intermediate

Sample Name	Sex	Strain	BioSample	BioProject
0822-G3-UCMI-01	female	UC Irvine	SAMN43850567	PRJNA1163388
0822-G3-UCMI-02	female	UC Irvine	SAMN43850568	PRJNA1163388
0822-G3-UCMI-03	female	UC Irvine	SAMN43850569	PRJNA1163388
0822-G3-UCMI-04	female	UC Irvine	SAMN43850570	PRJNA1163388
0822-G3-UCMI-05	female	UC Irvine	SAMN43850571	PRJNA1163388
0822-G3-UCMI-06	female	UC Irvine	SAMN43850572	PRJNA1163388
0822-G3-UCMI-07	female	UC Irvine	SAMN43850573	PRJNA1163388
0822-G3-UCMI-08	female	UC Irvine	SAMN43850574	PRJNA1163388
0822-G3-UCMI-09	female	UC Irvine	SAMN43850575	PRJNA1163388
0822-G3-UCMI-10	female	UC Irvine	SAMN43850576	PRJNA1163388
0822-G3-UCMI-11	female	UC Irvine	SAMN43850577	PRJNA1163388
0822-G3-UCMI-12	female	UC Irvine	SAMN43850578	PRJNA1163388
0822-G3-UCMI-13	female	UC Irvine	SAMN43850579	PRJNA1163388
0822-G3-UCMI-14	female	UC Irvine	SAMN43850580	PRJNA1163388
0822-G3-UCMI-15	female	UC Irvine	SAMN43850581	PRJNA1163388
0822-G3-UCMI-16	female	UC Irvine	SAMN43850582	PRJNA1163388
0822-G3-UCMI-17	female	UC Irvine	SAMN43850583	PRJNA1163388
0822-G3-UCMI-18	female	UC Irvine	SAMN43850584	PRJNA1163388
0822-G3-UCMI-19	female	UC Irvine	SAMN43850585	PRJNA1163388
0822-G3-UCMI-20	female	UC Irvine	SAMN43850586	PRJNA1163388
TN_18	female	Imperial College	SAMN07460669	PRJNA397539
TN_17	female	Imperial College	SAMN07460668	PRJNA397539
TN_16	female	Imperial College	SAMN07460667	PRJNA397539
TN_15	female	Imperial College	SAMN07460666	PRJNA397539
TN_14	female	Imperial College	SAMN07460665	PRJNA397539
TN_13	female	Imperial College	SAMN07460664	PRJNA397539
TN_12	male	Imperial College	SAMN07460663	PRJNA397539
TN_11	male	Imperial College	SAMN07460662	PRJNA397539
TN_20	female	Imperial College	SAMN07460671	PRJNA397539
TN_19	female	Imperial College	SAMN07460670	PRJNA397539
TN_06	male	Imperial College	SAMN07460657	PRJNA397539
TN_05	male	Imperial College	SAMN07460656	PRJNA397539
TN_08	male	Imperial College	SAMN07460659	PRJNA397539
TN_07	male	Imperial College	SAMN07460658	PRJNA397539
TN_02	male	Imperial College	SAMN07460653	PRJNA397539
TN_01	male	Imperial College	SAMN07460652	PRJNA397539
TN_04	male	Imperial College	SAMN07460655	PRJNA397539
TN_03	male	Imperial College	SAMN07460654	PRJNA397539
TN_10	male	Imperial College	SAMN07460661	PRJNA397539
TN_09	male	Imperial College	SAMN07460660	PRJNA397539
TN_23	female	Imperial College	SAMN07460674	PRJNA397539
TN_24	female	Imperial College	SAMN07460675	PRJNA397539
TN_21	female	Imperial College	SAMN07460672	PRJNA397539
TN_22	female	Imperial College	SAMN07460673	PRJNA397539
