# Supplementary material for: Defining the genetics of the widely used G3 strain of the mosquito, Anopheles gambiae
Source: Sci Rep. 2025 Apr 16;15:13142. doi: 10.1038/s41598-025-96391-y (PMC12003814; doi:10.1038/s41598-025-96391-y)
Supplement: Supplementary file 2 — Supplementary Material 2 [file 41598_2025_96391_MOESM2_ESM.docx]

**Supplementary Material**

**Supplementary Figure 1.** **Scree plot for principal component analysis.** Scree plots corresponding to the PCAs shown in Figure 2. The x-axis represents the first 50 PCs, while y-axis indicates the proportion of variance explained by each component.

**Supplementary Figure 2. Ancestry informative markers (AIM) genotypes.** A set of 700 SNPs from Ag1000G (phase 2) analysis between *An. gambiae* and *An. coluzzii*, where one allele is at or near fixation in one species, and an alternative allele in the other. This set of 122 samples was field collected in 5 countries in West Africa: Burkina Faso (*N*=1), Guinea (*N*=2), Guinea-Bissau (*N*=73), Mali (*N*=1), The Gambia (*N*=45). They were defined as intermediate between the two species by Ag1000G.

**Supplementary Table S1** - Pairwise F_ST_s using SNPs on chromosome 3 between G3 strain and natural populations of *An. gambiae* and *An. coluzzii* across Africa.

**Supplementary Table S2** - Pairwise F_ST_s using SNPs on chromosome X between G3 strain and natural populations of *An. gambiae* and *An. coluzzii* across Africa.

**Supplementary Table S3** – Ancestry Informative Markers (AIMs) between *An. gambiae* and *An. coluzzii*.

**Supplementary Table S4** – Metadata of subset of samples from Ag1000G used in this study, and sample ID and accession numbers of G3 strains.
